# Oncogenic CMTM6 drives M2a macrophages formation and fuels cervical cancer progression

**DOI:** 10.3389/fimmu.2025.1621816

**Published:** 2025-07-21

**Authors:** Bo Yin, Chun Chen, Baoyou Huang, Jianyi Ding, Haoran Hu, Huijuan Zhou, Yashi Zhu, Tiefeng Huang, Xiang He, Yuan Lu, Lingfei Han

**Affiliations:** ^1^ Department of Gynecology, Shanghai Key Laboratory of Maternal Fetal Medicine, Shanghai Institute of Maternal-Fetal Medicine and Gynecologic Oncology, Shanghai First Maternity and Infant Hospital, School of Medicine, Tongji University, Shanghai, China; ^2^ Department of Gynecology, The Obstetrics and Gynecology Hospital of Fudan University, Shanghai, China; ^3^ Department of Gynecology, The First Affiliated Hospital of Wenzhou Medical University, Wenzhou, Zhejiang, China; ^4^ Department of Gynecology, Shanghai Tenth People’s Hospital, Tongji University School of Medicine, Shanghai, China; ^5^ Department of Dermatology, Shuguang Hospital Affiliated to Shanghai University of Traditional Chinese Medicine, Shanghai, China

**Keywords:** cervical cancer, CMTM6, exosomal CMTM6, macrophage polarization, M2A

## Abstract

**Introduction:**

CMTM6, a member of the CKLF like MARVEL transmembrane (CMTM) gene family, has emerged as a critical orchestrator of oncogenic processes, yet its specific role in cervical cancer (CC) remains insufficiently characterized. Mounting evidence implicates that CMTM6 in sculpting an immunosuppressive tumor microenvironment (TME).

**Methods:**

We investigated the expression and functional role of CMTM6 in CC cells using in vitro biological assays and a mouse xenograft model. The impact of CMTM6 on macrophage polarization and its association with tumor progression were systematically evaluated through a series of in vitro and in vivo experiments, focusing on the induction of M2a macrophage polarization and activation of the mTOR signaling pathway.

**Results:**

Our results demonstrate that exosomes secreted by CC cells encapsulate CMTM6, which is actively internalized by macrophages, inducing M2a polarization and triggering immunosuppressive pathways. Excessive macrophage infiltration in the TME, particularly in the presence of CMTM6, is strongly associated with unfavorable prognosis. Furthermore, exosomal CMTM6 activates the mTOR signaling pathway in tumor-associated macrophages, enhancing CCL2 secretion, which further promotes M2a polarization and accelerates tumor metastasis.

**Discussion:**

These findings highlight exosomal CMTM6 as a crucial driver of immune suppression in CC, with the CMTM6/CD206/CCL2 axis significantly increasing the risk for CC patients. Our study underscores the potential of exosomal CMTM6 as both a prognostic biomarker and a therapeutic target for CC immunotherapy.

## 1 Introduction

Cervical cancer (CC) is recognized as one of the most prevalent malignancies affecting women worldwide, and it stands as a major cause of female mortality (1). Notably, the widespread implementation of routine CC screening has led to a significant improvement in the detection of early-stage cases, thus enhancing patient prognoses. However, conventional approaches such as surgery and radiation therapy exhibit limited efficacy when the disease progresses to advanced stages involving recurrence and metastasis (2). Incidentally, research has highlighted that the infiltration and interplay of immune cells within the tumor tissue and immune microenvironment play a critical role in the clinical outcomes of patients with CC (3). Consequently, immunotherapy has emerged as a promising treatment strategy, offering the potential to enhance survival rates in these individuals. In pursuit of assessing the inherent potential of immunotherapy for CC, it becomes imperative to delve into the intricate interactions between tumor cells and immune cells within the tumor microenvironment (TME). This enhanced understanding of their interplay holds promising implications for developing targeted and tailored immunotherapeutic strategies for individual patients.

Macrophages, as key regulators in cancer progression, comprise the largest proportion of tumor-infiltrating immune cells. Macrophages are generally categorized into two distinct subtypes: classically activated M1 macrophages and alternatively activated M2 macrophages (4). Infiltrating macrophages predominantly exhibit an M2 phenotype, commonly referred to as tumor–associated macrophages (TAMs) in TME (5). TAMs are essential for promoting tumor cell proliferation, metastasis, and angiogenesis. Furthermore, tumor-secreted exogenous factors, such as exosomes, enhance the chemotaxis and migration of monocytes as well as their differentiation within the TME (6–8). Exosomes, ranging in size from 30–150 nm, exert their effects of cellular function through autocrine or paracrine pathways carrying bioactive molecules such as proteins, nucleic acids, and lipids, which reshape the TME and accelerate tumor progression, potentially impeding immunotherapy (9). For instance, exosomes within the TME inhibit the activation of CD8^+^ T cells, consequently leading to a reduced response to anti-programmed death ligand 1 (PD-L1) therapy (10). TAMs, which have significant functions in the TME, can be directed by exosomes to adopt an immunosuppressive phenotype associated with poor prognosis. Currently, the mechanisms underlying the activation of M2 macrophages by tumor cells remain elusive in CC.

CKLF-like MARVEL transmembrane 6 (CMTM6), a transmembrane protein, evinces complicated and opposing roles in the realm of tumorigenesis. Notably, in select tumor types, it manifests tumor suppressive properties by curbing malignant growth and metastatic dissemination. However, this duality becomes apparent in immunotherapy-resistant tumors, where heightened expression of CMTM6 is a telltale sign of tumor resistance and unfavorable prognosis (11, 12). Within our research of CC, a fascinating observation ensued, revealing that CMTM6 proficiently instigates the autonomous progression of tumor cells through an autocrine mechanism. This provocation is accompanied by the orchestration of M2a macrophage programming facilitated by the exosomal CMTM6 secretion. Overall, these pioneering study outcomes anticipate aiding researchers in identifying potential, novel biomarkers specific to CC, while concurrently paving the way for the development of innovative strategies that accurately predict an individual’s susceptibility to CC.

## 2 Materials and methods

### 2.1 Clinical specimens and ethical approval

CC tissue and normal cervical samples were collected from individuals who underwent surgical resection at Shanghai First Maternity and Infant Hospital between the time span of May 2020 and June 2023. All clinicopathological diagnoses were confirmed by a minimum of two pathologists. None of the patients received chemotherapy or radiotherapy prior to the surgery. Immediately after resection, all tissue samples were promptly preserved in liquid nitrogen and stored in a freezer at a temperature of -80**°**C until further analysis. The study was granted approval by the Ethics Committee of the Shanghai First Maternity and Infant Hospital, with all participating patients having provided informed consent by signing the necessary forms.

### 2.2 Cell culture and treatment

HEK 293T, THP-1, RAW264.7, and CC cell lines (TC-1, HeLa, SiHa, Caski, C33A and ME180) were obtained from the Cell Banks of the Type Culture Collection of the Chinese Academy of Sciences (Shanghai, China), while Caski, C33A, ME180 and TC-1 cell lines were purchased from FuHeng Biology (Shanghai, China). Cells were cultured in DMEM (Gibco, USA) for HEK 293T, RAW264.7, and CC cell lines, and RPMI 1640 medium (Gibco, USA) for THP-1, in a 37°C, 5% CO2 humidified incubator. All media were supplemented with 10% fetal bovine serum (C9050, NCM Biotech, China) and 1% penicillin/streptomycin.

### 2.3 RNA extraction, reverse transcription, and qPCR

Total RNA was extracted from cells or tissues by using RNAiso Plus reagent (Takara, Japan) in accordance with the instructions. The RNA was reverse-transcribed to cDNA using ABScript II RT Master Mix (RK20429, ABclonal, China). qPCR was performed using Genious 2× SYBR Green Fast qPCR Mix (RK21203, ABclonal, China) according to the manufacturer’s protocols. Target gene expression was standardized to GAPDH expression. The primer sequences are listed in **Supplementary Table S1**.

### 2.4 Antibodies and reagents

The antibodies and reagents used in this study were shown in **Supplementary Table S2**.

### 2.5 Western blotting

In brief, cell or exosome samples were collected using standard RIPA buffer (WB3100, NCM Biotech, China) containing protease and phosphatase inhibitor cocktails (P002, NCM Biotech, China). WB analyses were conducted according to the previously described method (13). The quantification of protein bands was performed using Image J software (USA).

### 2.6 Immunohistochemistry

Tissue sections were dewaxed with xylene and dehydrated with different gradient alcohol series. After that, the slices were treated with 3% hydrogen peroxide and pressed boiled to extract the antigens. The slices were then incubated with antibodies overnight at 4°C. Then incubated with the corresponding secondary antibodies at room temperature (25°C) for 1h. Immunostaining is performed using 3’-diaminobenzidine (DAB) according to the manufacturer’s instructions. Finally, the slices were stained with hematoxylin and sealed with neutral gum.

### 2.7 Cell transfection

The CMTM6 small interfering RNAs (siRNAs) were provided from Hanbio Biotechnology Co., Ltd (Shanghai, China) and Lipofectamine™ 3000 Transfection Reagent (Thermo Fisher Scientific, USA) was used to transfect the siRNAs into targeted cells according to the manufacturer’s instructions. 12 h after transfection, the cell culture medium was replaced with a fresh medium for subsequent experiments. All sequences used in this study are available in **Supplementary Table S3**.

### 2.8 Vectors construction and construction of stable cell lines

For shRNA work, the designed Oligo (Youbio, Changsha, China) was annealed to form a double-stranded chain, while the carrier pLKO.1 was digested by EcoRI and AgeI enzymes. The annealed product was then connected to the carrier. The synthesized shRNA plasmid, psPAX2, and pMD2.G were all simultaneously transferred into 293T cells to synthesize the virus. The virus was then transfected into the target cells, which were subsequently screened with 4ug/ml puromycin for 48h. The TC-1 cells with stable gene knockout were harvested. Non-targeted shRNA was used as control.

For CRISPR/Cas9 knockout system, lentiCRISPRv2 was utilized as a vector and digested with FastDigest enzyme. The remaining steps followed the same procedure as shRNA work. The sgRNA were designed using the MIT online tool (http://crispr.mit.edu). The effectiveness of gene depletion was assessed through WB. All sequences used in this study are available in **Supplementary Table S3**.

### 2.9 Colony formation assay

The cells under investigation were seeded into individual wells of a 6-well plate at a density of 1,000 or 2,000 cells per well. Following a 10-day incubation period, the cells were immobilized using a 4% paraformaldehyde fixative for a duration of 20 min. Subsequently, the cells were stained with a 0.5% crystal violet solution for 20 min. Finally, the number of clonally derived cells was quantified.

### 2.10 Apoptosis assay

Following the digestion with EDTA-free trypsin, the cells intended for analysis were treated with annexin V-FITC and PI fluorescence (40302ES08, Yeason Biotechnology, Shanghai) staining and incubated in 100μL of binding buffer for 30 min in a light-free environment. Flow cytometry was employed to capture fluorescence data, with a total of 1 × 10^4^ cells recorded per sample.

### 2.11 Transwell assays

The migratory and invasive capabilities of CC cells were assessed using 24-well transwell plates (Corning, USA) equipped with an 8.0μm pore polycarbonate membrane. For the invasion assay, the upper surface of the membrane was pre-coated with Matrigel mix (D23016-0010, D1 Medical Technology, Hangzhou, China), while for the migration assay, no coating was applied. Tumor cells were seeded in the upper chamber using 200 µL of serum-free DMEM medium, while the bottom chamber was supplemented with 600 µL of medium containing 10% FBS. After a 24-h incubation period, the cells remaining in the upper chamber were gently removed, and the cells that had either migrated through the membrane or invaded it were fixed with paraformaldehyde and stained using a 0.1% crystal violet solution. Images were captured using an inverted microscope, and cell numbers were quantified utilizing the ImageJ software (USA).

### 2.12 Hematoxylin and eosin staining

The lung tissues were fixed in paraffin and cut into 4 µm sections. Hematoxylin and eosin (H&E) staining was performed using the H&E Staining Kit (SD6146, SIMUWU, China) according to the manufacturer’s instructions.

### 2.13 Flow cytometry

Specimens were prepared according to the previously described method (3). Labeled cells were analyzed on a BD FACSCalibur or BD FACSCanto™ II flow cytometer and the data were processed using FlowJo software (USA).

### 2.14 Macrophage culture and treatment

Macrophages were transformed from THP-1 cells. Cells were cultured in RPMI 1640 medium (Gibco, USA) supplemented with 10% FBS at 37°C in a humidified 5% CO_2_ atmosphere. To transform the cells into macrophages(M0), THP-1 monocytes were cocultured with 100 ng/mL phorbol 12-myristate 13-acetate (PMA, HY18739, MedChemExpress, China) in RPMI 1640 medium without FBS for 48 h. Macrophages treated with 20 ng/ml interleukin 4 (IL-4) (HY-P70445, MedChemExpress, China) for 24 h were polarized into M2 phenotype (6). To investigate the effects of exosomes on macrophages, 40μg of exosomes were added to the macrophage culture medium and incubated in the cell incubator for another 24 or 48 h prior to harvesting cells for subsequent experiments.

### 2.15 Macrophage phagocytosis assays

For the *in vitro* phagocytosis assay, 1×10^5^ macrophages were stained with the CD11b antibody and plated in transparent 96-well plates. Simultaneously, 1×10^5^ tumor cells were labeled with a 5μM green fluorescent dye, CFSE (BD Biosciences). After co-culturing macrophages with tumor cells for 4 h, FCM was employed for result analysis.

### 2.16 Exosome extraction and identification

Ultracentrifugation methods were applied to isolate exosomes from the supernatants of CC cells according to previously published protocol (14). In brief, the cells were cultured in a complementary medium until reaching approximately 80% confluence, Then the medium was replaced with a defined medium without FBS. After 2 days of culture, the supernatants were harvested and subjected to sequential centrifugations at 800 × g for 15 min, 2,000 × g for 15 min, and 10,000 × g for 60 min. The resulting supernatants were filtrated through a 0.22μm PVDF filter (Millipore, USA). The filtered supernatants were collected to isolate exosomes by ultracentrifugation using 120,000 × g for 2 h (Beckman Coulter). Temporarily unused exosomes were frozen at -80°C with PBS. For exosome identification, 50μL of exosomes resuspended in PBS were adsorbed onto a copper mesh with a polyvinyl methylacetate acetate support membrane, stained with 2% phosphotungstic acid, and observed for size using a transmission electron microscope (TEM). The particle size and concentration of exosomes were observed using nanoparticle tracking analysis (NTA).

### 2.17 Exosome uptake assay

To monitor exosomal trafficking, exosomes isolated from the culture medium were labeled with a Dil fluorescent cell linker kit (Thermo Fisher Scientific, USA). After Dil staining, the exosomes were washed in PBS and resuspended in PBS. Plus, the Dil-labeled exosomes were incubated with macrophages for 24 h. Nuclei and the cytoskeleton were stained with DAPI and FITC-phalloidin (40735ES75, Yeason Biotechnology, Shanghai), respectively. Finally, the uptake of exosomes was examined by confocal fluorescence microscopy.

### 2.18 Enzyme-linked immunosorbent assay

Secretion of CCL2 into the culture supernatant from macrophages were detected with the ELISA kits (YPG0179, UpingBioHANGZHOU, China) according to the manufacturer’s instructions.

### 2.19 Animal studies

All animal care and experiments were conducted in accordance with the guidelines stipulated by the National Institutes of Health and approved by the Animal Care Committee of Tongji University. C57BL/6 and Balb/c mice (6–8 weeks old) were purchased from Beijing Vital River Laboratory Animal Technology Company and settled in a specific pathogen-free environment. Mice were randomly allocated to groups and received a subcutaneous injection of 2 × 10^6^ mouse tumor cells (150μL) in the animal’s right flank.

In tumor metastasis assay *in vivo*, Balb/c mice were intravenously injected with tumor cells (2 × 10^6^ cells per mouse) via their tail veins. After an 8-week period, the mice were humanely sacrificed, and the number of metastatic foci was computed.

For the effects of exosomes on macrophages *in vivo*, the macrophages were pretreated with either PBS or exosomes derived from TC-1. Next, a mixture of tumor cells and macrophages in a 10-to-1 ratio was injected subcutaneously into C57BL/6 mice. A total of 4 intratumoral injections of either PBS or exosomes were administered before the mice were euthanized.

Tumor volumes were measured at fixed intervals and calculated as volume = width^2^ × length × 0.5. The tumor and lung tissues were fixed with paraformaldehyde for HE and IHC staining.

### 2.20 Quantification and statistical analysis

Statistical analyses were performed using R (v4.2.2) or GraphPad Prism (v.9) software. Cell counts were analyzed using the ImageJ software, and FCM data were quantified using the FlowJo (v10) software. Details are provided in the figure legends. A p-value of <0.05 was considered statistically significant.

## 3 Results

### 3.1 CMTM6 is overexpressed in CC and associated with weakened prognosis

**Figure 1:**
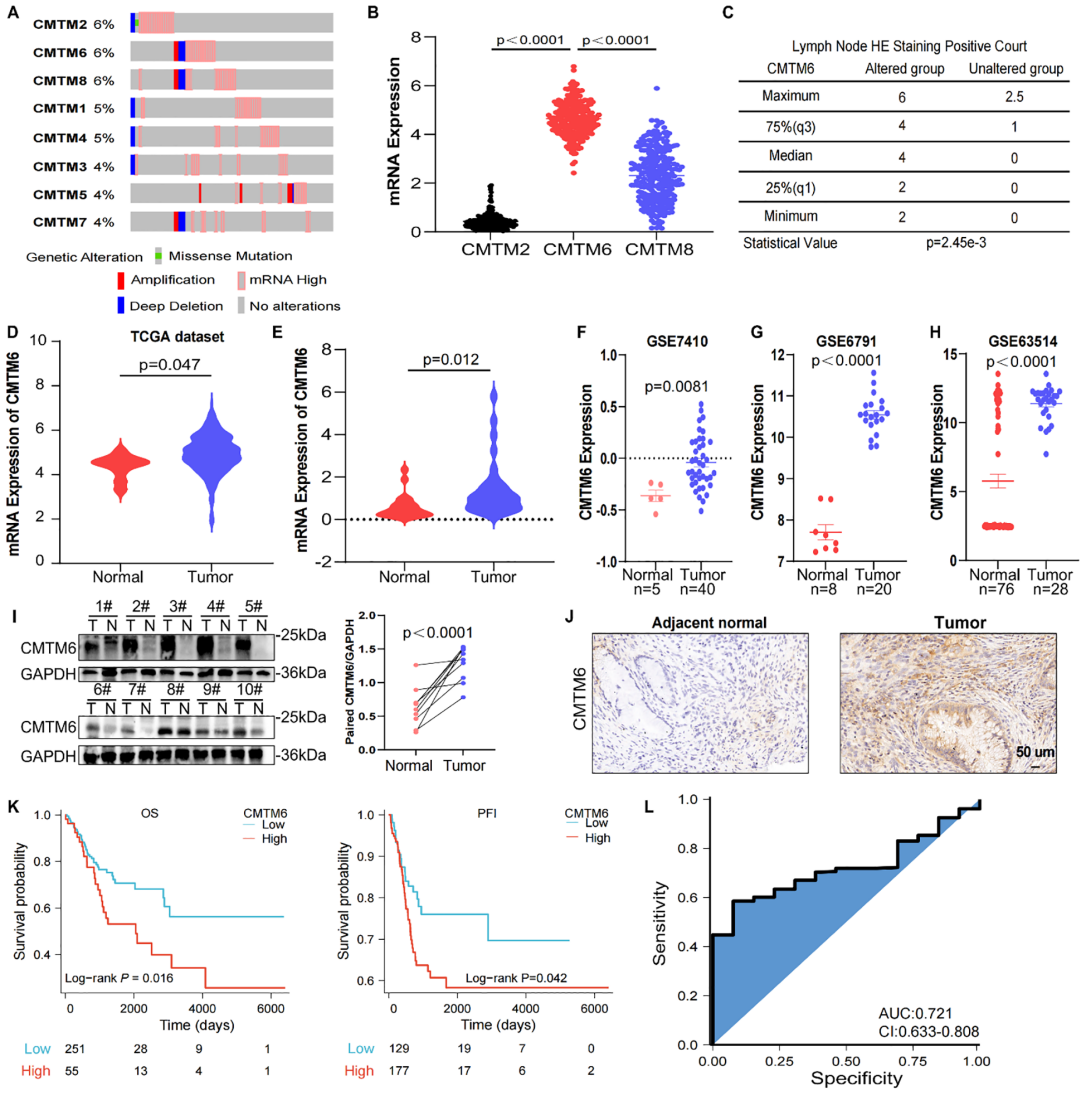
CMTM6 is upregulated and is associated with a dismal survival in CC. **(A)** DNA CNV of CMTM family genes in CC based on the TCGA dataset. **(B)** The mRNA expression of CMTM2, CMTM6 and CMTM8 were evaluated via TCGA-CESC. **(C)** The relationship between CMTM6 genetic alteration and positive HE staining in lymph nodes was analyzed by cbioportal database. **(D)** CMTM6 mRNA expression in CC and normal samples from TCGA dataset and GTEx database were analyzed. **(E)** The differences of CMTM6 mRNA levels of 30 CC tissues and 20 normal tissues across our clinical specimen were measured. **(F–H)** The analysis of CMTM6 mRNA expression in 3 GEO datasets (GSE7410, GSE6791, GSE63514). **(I)** WB analysis of CMTM6 protein expression in CC tissues and matched normal cervix tissues (left), n=10. Protein quantified (right) using ImageJ software. **(J)** Representative images of CMTM6 IHC staining in adjacent normal tissues and CC tissues were showed. **(K)** Kaplan–Meier curves revealed OS (left) and PFI (right) of CC patients with high CMTM6 *vs* low CMTM6 levels (based on best cut off). Analyzed using the log-rank test. **(L)** ROC curve analysis of the sensitivity, specificity and AUC for CMTM6 was calculated to be 0.721 in CC patients from TCGA data. For B, D, E, F, G, H, data are presented as the mean ± SEM, unpaired two-sided Student’s t-test. For I, unpaired two-sided Student’s t-test.

To investigate the potential role of CMTM family in CC, we initially analyzed the copy number variations (CNV) of CMTM1–8 in The Cancer Genome Atlas (TCGA) dataset as shown in **Figure 1A**. It was found that CMTM2/6/8 are typically amplified in CC and the amplification of these genes is correlated with increased mRNA expression. Further research showed a significant upregulation of CMTM6 compared to CMTM2 and CMTM8 (**Figure 1B**). Following that, we found that CMTM6 genetic alteration was linked with lymph node HE staining positive and advanced stage via cbioportal (https://www.cbioportal.org/) database (**Figure 1C**; **Supplementary Figure S1A-C**). In addition, the data from TCGA showed that CMTM6 expression in CC tissues was higher than that in normal tissues (**Figure 1D**). This is further confirmed by the cohort (30 CC tissues and 20 normal tissues) of Shanghai First Maternal and Infant Health Hospital and Gene Expression Omnibus (GEO: GSE7410, GSE6791, GSE63514) database (**Figures 1E–H**). The relationship between CMTM6 expression and a range of clinicopathological parameters was systematically evaluated and found that CMTM6 expression exhibited a significant positive correlation with both tumor size and stromal invasion. In contrast, no statistically significant associations were observed between CMTM6 expression and age, pathologic types, FIGO stage, lymph node metastasis, lymphovascular invasion, and vaginal involvement (**Table 1**). Then, WB and IHC analysis both revealed that CMTM6 expression was higher in tumor tissues than matched non-tumor tissues (**Figures 1I, J**). Finally, we analyzed the relationship between CMTM6 expression levels and patient prognosis using the TCGA cohort and/found that higher CMTM6 expression was positively associated with poorer overall survival (OS) and progression-free interval (PFI) (**Figure 1K**). To assess the diagnostic value of CMTM6 in CC, a receiver operating characteristic (ROC) curve was generated, and the area under the curve (AUC) for CMTM6 was calculated to be 0.721 (**Figure 1L**), suggesting that CMTM6 mRNA expression may serve as a predictive factor for CC progression. Collectively, CMTM6 probably serve as a promising diagnostic biomarker for CC patients and its upregulation linked with inferior survival.

**Table 1:** Correlation between the CMTM6 levels and clinicopathological features in 30 CC patients.

Characteristics	Total	High expression (15)	Low expression(15)	P-value
Age(year)				0.99
>45	20	10 (66.7%)	10 (66.7%)	
≤45	10	5 (33.3%)	5 (33.3%)	
Pathologic types				0.99
Squamous carcinoma	24	12 (80%)	12 (80%)	
Adenosquamous and adenocarcinoma	6	3 (20%)	3 (20%)	
FIGO stage (2009)				0.71
I	13	7 (46.7%)	6 (40%)	
II+III	17	8 (53.3%)	9 (60%)	
Tumor size				0.025*
≤4 cm	18	6 (40%)	12 (80%)	
>4 cm	12	9 (60%)	3 (20%)	
Lymph node metastasis				0.26
No	19	8 (53.3%)	11 (73.3%)	
Yes	11	7 (46.7%)	4 (26.7%)	
Lymphovascular invasion				0.72
No	15	7 (46.7%)	8 (53.3%)	
Yes	15	8 (53.3%)	7 (46.7%)	
Stromal invasion				0.02*
≤1/2	11	2 (13.3%)	8 (53.3%)	
>1/2	19	13 (86.7%)	7 (46.7%)	
Vaginal involvement				0.99
No	16	8 (53.3%)	8 (53.3%)	
Yes	14	7 (46.7%)	7 (46.7%)	

Chi-square test. * represents p < 0.05.

### 3.2 CMTM6 stimulated CC development *in vitro* and *in vivo*

**Figure 2:**
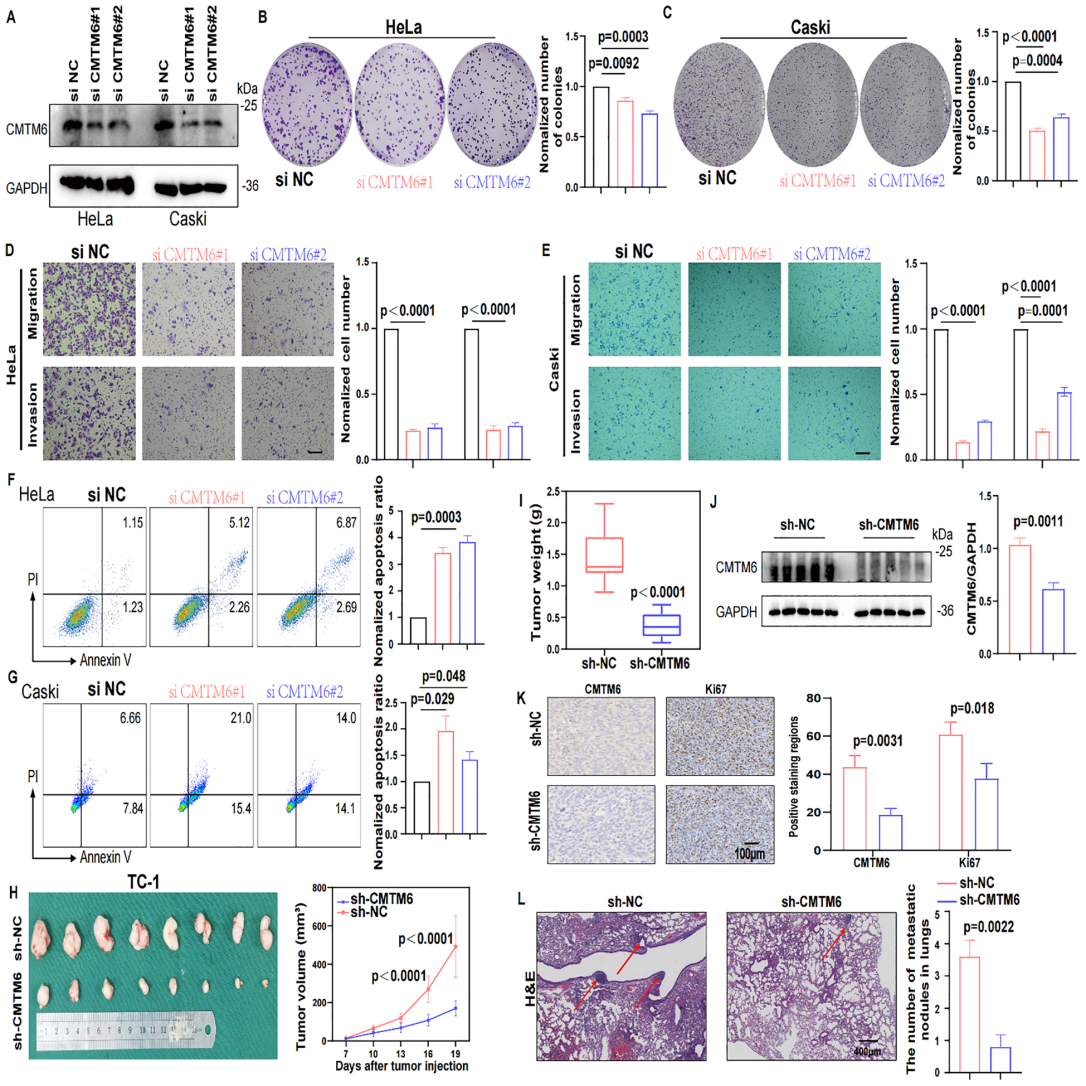
CMTM6 stimulated CC development *in vitro* and *in vivo.* **(A)** WB showed that the protein knockout efficiency of CMTM6 siRNA on HeLa and Caski. **(B, C)** The proliferation of HeLa **(B)** and Caski **(C)** cells were evaluated when CMTM6 of cells was knockdown. **(D, E)** The migration and invasion of HeLa **(D)** and Caski **(E)** when CMTM6 protein expression downregulated were examined using Transwell assays. All panels are the same magnification. Scale bar, 100μm. **(F, G)** The apoptosis of HeLa **(F)** and Caski **(G)** when CMTM6 protein expression decreases were studied by FCM, respectively. **(H)** The images of xenografts and tumor growth of the TC-1 cells (sh-NC versus sh-CMTM6) in C57BL/6 mice (n=8 mice per group) and tumor growth curves of two groups were recorded. **(I)** TC-1-tumor weights were calculated between groups. **(J)** WB detected CMTM6 protein expression in the TC-1-tumors between groups (left), protein quantified (right) using ImageJ software (right). **(K)** The CMTM6 and proliferation index Ki-67 was evaluated by IHC in aforementioned tumor-bearing mice (left) and quantification of the percentage of CMTM6 and Ki-67 positive staining areas in tumor-bearing tissues (right) (n=3). All panels are the same magnification. Scale bar, 100μm. **(L)** Representative H&E-stained lung tissues and quantification of metastatic nodules in recipient mice following tail vein injection of TC-1 cells (sh-NC versus sh-CMTM6). All panels are the same magnification. Scale bar, 400μm. For **A**, the representative WB images were shown, n=3 independent experiments per group. For **(B–E)** data are presented as the mean ± SD, unpaired two-sided Student’s t-test, data are shown as normalized to the NC group which was set to 1 after normalization. n=3 independent experiments per group. For (**F, G)** the representative FCM results were shown, data are presented as the mean ± SD, unpaired two-sided Student’s t-test, data are shown as normalized to the NC group which was set to 1 after normalization, n=3 independent experiments per group. For **(H, I)** data are presented as the mean ± SEM, unpaired two-sided Student’s t-test, n=8 per group. For **(J)** data are presented as the mean ± SEM, unpaired two-sided Student’s t-test, n=5 per group. For **(K)** data are presented as the mean ± SD, unpaired two-sided Student’s t-test, n=3. For **(L)** data are presented as the mean ± SD of three independent experiments, unpaired two-sided Student’s t-test.

To explore the biological effects of CMTM6 in CC, we firstly detected CMTM6 expression in CC cell lines (**Supplementary Figure S2A**). Therefore, we transfected CMTM6 siRNA (defined as siCMTM6#1 and siCMTM6#2, respectively) into HeLa and Caski cell lines. WB analysis exhibited that siRNAs obviously suppress CMTM6 protein expression (**Figure 2A**). We also constructed CMTM6 knockdown TC-1 cell—a murine-derived cell line expressing HPV16 E6 and E7—with two shRNAs (named as sh-CMTM6–1 and sh-CMTM6-2), with sh-NC as the control (**Supplementary Figure S2B**). Compared to the control, silencing CMTM6 impaired the clonogenicity of CC cells (**Figures 2B, C** **;** **Supplementary Figure S2C**). Consistently, Transwell assay revealed that downregulation of CMTM6 expression remarkedly impaired the migration and invasion behavior in CC cells (**Figure 2D, E** **;** **Supplementary Figures S2D, E**). As expected, the down-regulation of CMTM6 enhanced the apoptosis ability of CC cells (**Figures 2F, G** **;** **Supplementary Figure S2F**). Given the aforementioned findings, we examined the influence of CMTM6 of CC *in vivo* employing TC-1 tumor- bearing mice and lung metastasis model. The results disclosed that CMTM6 knockdown undermined tumor proliferation (**Figures 2H, I**). Afterwards, the WB analysis indicated CMTM6 low-expression in tumor tissues and IHC showed that CMTM6 knockdown inhibited the Ki67 expression (**Figures 2J, K**). In the lung metastasis model, CMTM6 knockdown markedly reduced both the number and size of metastatic nodules. Metastatic lung tissues showed severe disruption of alveolar architecture, enlarged pleomorphic tumor cell nuclei, and abundant neovascularization (**Figure 2L**). Generally speaking, these discoveries indicate that CMTM6 exerts an oncogenic role in CC.

### 3.3 CMTM6 promotes CC progression by modulating the immune microenvironment through macrophages

**Figure 3:**
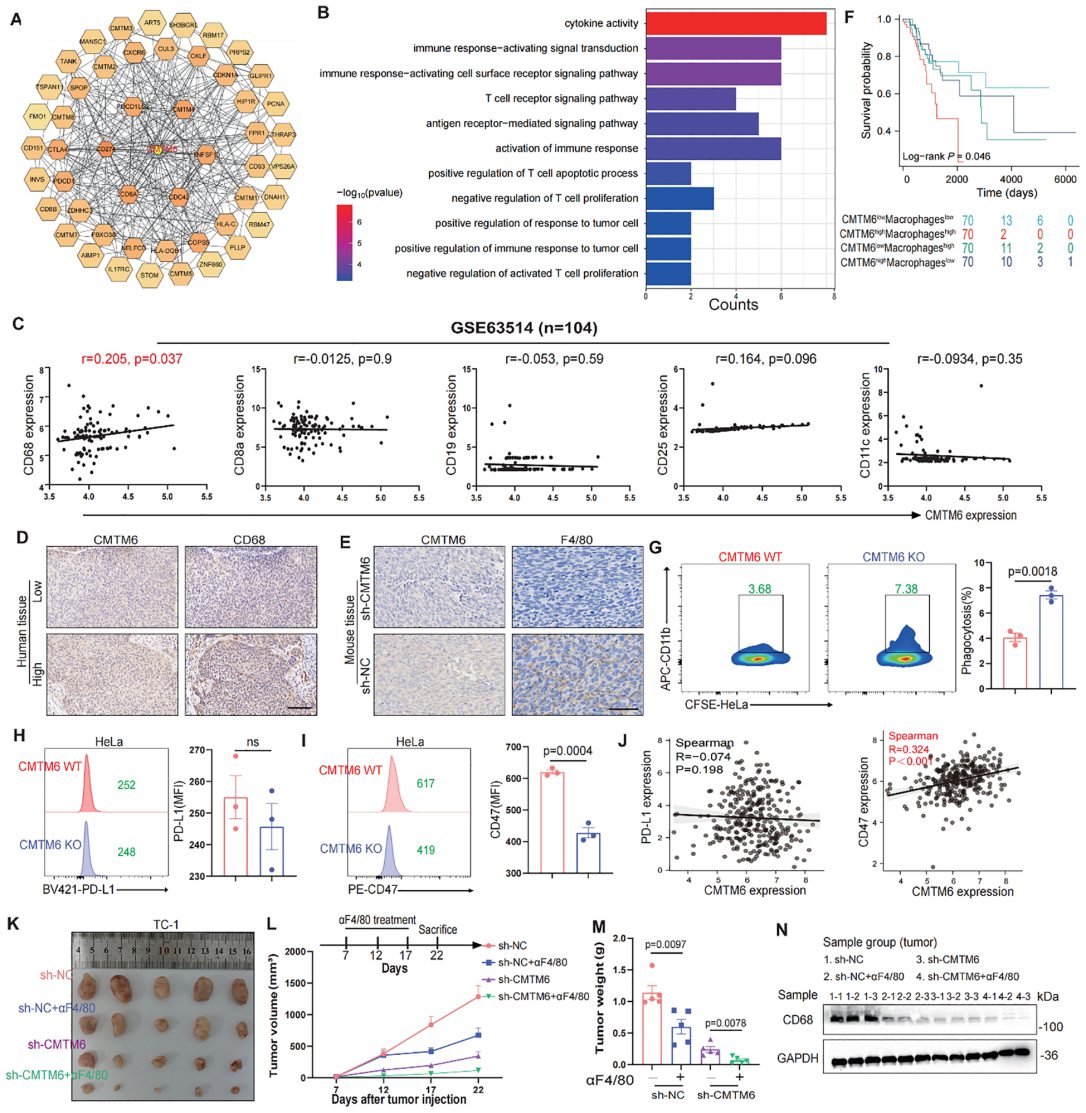
CMTM6 promotes CC progression by modulating the immune microenvironment through macrophages. **(A)** Schematic diagram of interactors of CMTM6. **(B)** GO enrichment analysis of CMTM6 interactors. **(C)** Spearman Correlation analysis of CD68^+^ macrophages, CD8^+^ T cells, CD19^+^ B cells, CD25^+^ Tregs and CD11c^+^ DCs infiltration and the CMTM6 mRNA expression in GSE63514 (n=104). **(D, E)** Representative images showed IHC staining of CD68 or F4/80 and CMTM6 in the same field of human CC tissues **(D)** and mouse tumors **(E)**. All panels are the same magnification. Scale bar, 100μm. **(F)** CMTM6 mRNA expression and macrophage infiltration as markers for prediction of OS in TCGA-CESC cohort. Data were classified into CMTM6-high/CMTM6-low and high macrophage/low macrophage signature. Analyzed using the log-rank test. **(G)** The phagocytic activity of macrophages toward HeLa cells following CMTM6 downregulation was assessed by FCM. **(H, I)** FCM was used to assess the changes in PD-L1 **(H)** and CD47 **(I)** expression levels following CMTM6 downregulation in HeLa cells. **(J)** The correlation between CMTM6 and PD-L1 and CD47 mRNA expression was analyzed in GEPIA database. **(K)** TC-1 cells (sh-NC versus sh-CMTM6) were implanted into C57BL/6 mice (four groups, five mice per group). Among these, two groups received antibody treatment, and tumors were excised at designated time points. **(L)** Starting on day seven post-tumor implantation, mice were administered intraperitoneal αF4/80 injections every five days for a total of three doses. Tumor volume was measured every five days, and on day 22, the mice were humanely euthanized. **(M)** Tumor weights of each group were shown. **(N)** WB showing the protein expression of CD68 in tumor tissues of each group (n=3). For **(G)** data are presented as the mean ± SD, unpaired two-sided Student’s t-test, n=3 independent experiments per group. For **(H, I)** data are presented as the mean ± SD, unpaired two-sided Student’s t-test, n=3 independent experiments per group, the data are presented as the MFI values. For **(L, M)** data are presented as the mean ± SEM, n=5, one-way ANOVA followed by Tukey multiple comparison test.

To further investigate the oncogenic role of CMTM6 in CC, we firstly queried the STRING database to identify 50 interactors of CMTM6 (**Figure 3A**). Then we performed enrichment analysis on these interactors, and GO results showed they are significantly linked to immune regulation, including cytokine activity and immune response signaling. (**Figure 3B**; **Supplementary Figures S3A, B**). Building on these results, we speculated that CMTM6 might play a role in shaping the tumor immune microenvironment in CC. Hence, we first analyzed the relationship between CMTM6 expression and tumor-infiltrating immune cells using the GEO dataset GSE63514 (n = 104). The results showed that CMTM6 expression was significantly correlated with increased infiltration of CD68-positive macrophages, whereas the correlations with markers of CD8^+^ T cells (CD8A), B cells (CD19), regulatory T cells (Tregs, CD25), dendritic cells (DCs, CD11c), and neutrophils (LY6G) were relatively weak and not statistically significant. Similarly, the correlation with markers of myeloid-derived suppressor cells (MDSCs) was also limited (**Figure 3C**; **Supplementary Figure S3C**). Collectively, the data suggest that CMTM6 may be specifically associated with the infiltration of TAMs in the CC microenvironment. Moreover, IHC demonstrated that heightened CMTM6 in human tissues was closely linked to a marked augmentation in macrophage infiltration, a trend that was also observed in cases with TC-1 mouse tumors (**Figures 3D, E**). These results indicate that CMTM6 is associated with enhanced macrophage infiltration, suggesting its potential role in modulating macrophage-mediated immune responses in CC.

To further validate our findings, we stratified the TCGA-CESC cohort into four groups based on CMTM6 expression and macrophage infiltration levels. Survival analysis revealed that patients with high CMTM6 expression and increased macrophage infiltration had the poorest prognosis (**Figure 3F**). Macrophages are key to phagocytosis, a vital defense mechanism. Tumor cells exploit immune checkpoint molecules (ICMs), like PD-L1 and CD47, to escape macrophage-mediated surveillance (15). To verify our hypothesis, we conducted *in vitro* macrophage phagocytosis assays. The results proved that macrophages engulfed more CMTM6-KO cells compared to the control cells (**Figure 3G**, **Supplementary Figures S3D–F**). FCM results exposed a significant downregulation of CD47 expression in CMTM6-KO CC cells, but PD-L1 expression was not affected (**Figures 3H, I**; **Supplementary Figure S3G, H**). Likewise, the findings from the TCGA-CESC reinforce this (**Figures 3J**). To assess whether the suppression of tumor growth following CMTM6 knockdown is attributable to a reduction in macrophage-mediated effects, we employed anti-F4/80 (α-F4/80) antibody to deplete macrophages in the TC-1 tumor model. Macrophage depletion alone produced a tumor-inhibitory effect comparable to that observed with CMTM6 knockdown. Notably, the most pronounced attenuation of tumor progression occurred when CMTM6 knockdown was combined with macrophage depletion (**Figures 3K–M**). WB results also showed down-regulated protein expression of CD68 in the tumor-bearing mice (**Figure 3N**). These findings suggest that the absence of CMTM6 in the tumor impairs the infiltration of TAMs and reduces oncogenesis.

### 3.4 CMTM6 drives M2a malignant phenotype in macrophages via exosomes and mTOR activation

**Figure 4:**
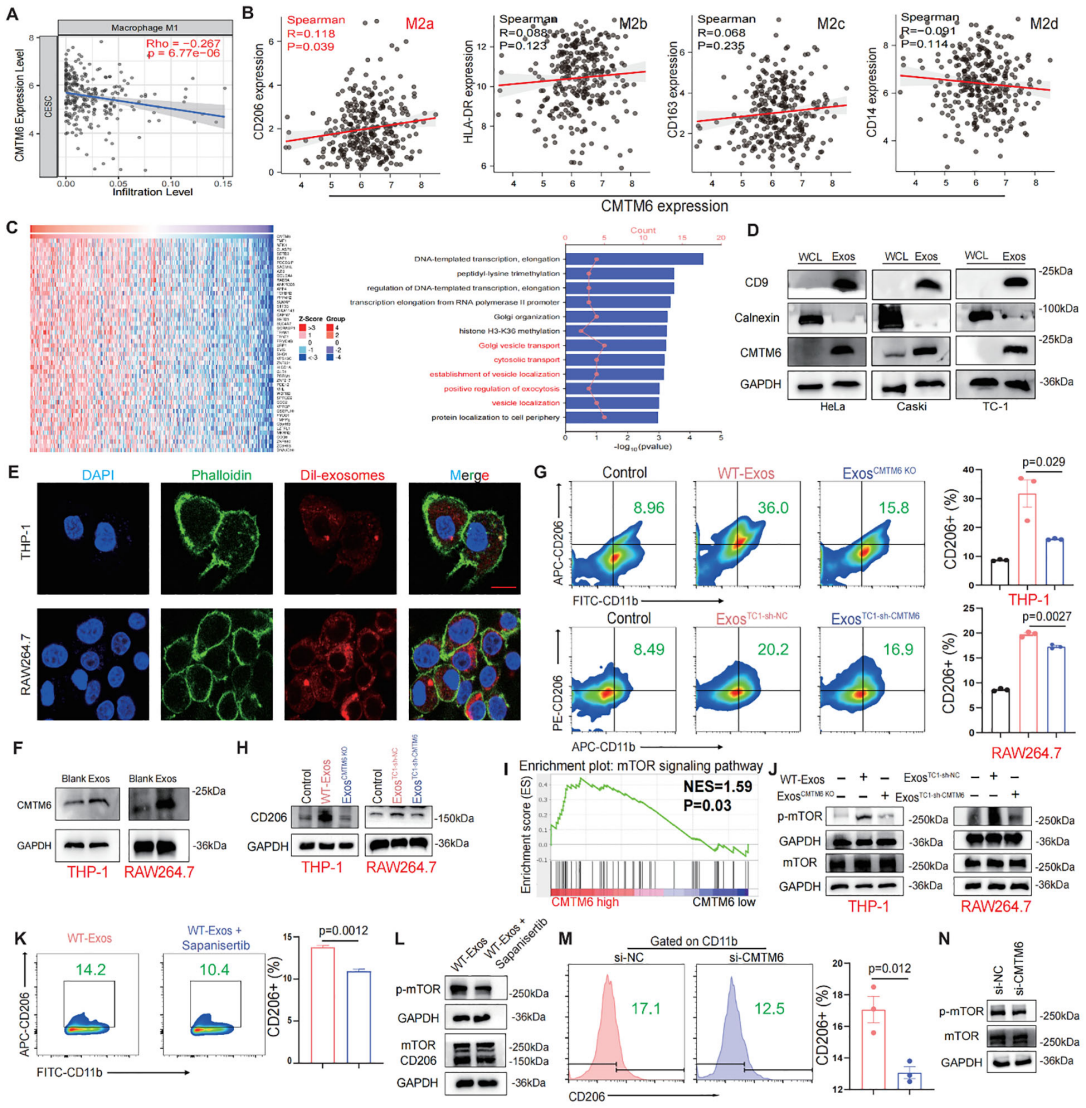
CMTM6 drives M2a malignant phenotype in macrophages via exosomes and mTOR activation. **(A)** Correlation analysis for CMTM6 and M1macrophage infiltration by TIMER databases. **(B)** Correlation analysis CMTM6 and CD206 (M2a), HLA-DR (M2b), CD163 (M2c), CD14 (M2d) by TCGA-CESC cohort. **(C)** Heat maps of genes positively associated with CMTM6 expression in the TCGA-CESC cohort (left) and GO analysis of these genes (right). **(D)** WB detection of CMTM6 protein expression in CC cells and their extracted exosomes. **(E)** Representative fluorescence microscopy images illustrated the process of CC cell-derived exosomes transmitted to macrophages. Scale bar, 25μm. **(F)** The effects of CC cell derived exosomes on CMTM6 protein expression after being internalized by macrophages were analyzed by WB. **(G)** FCM determined the proportion of human and mouse M2a (CD11b^+^CD206^+^) macrophages (THP-1 and RAW264.7) treated by CC cell-derived exosomes. **(H)** The protein levels of CD206 of macrophages educated by CC-derived exosomes were analyzed by WB. **(I)** Single-gene GSEA analysis of CMTM6 in the TCGA cohort identified significant enrichment in the mTOR signaling pathway. **(J)** WB analysis was used to assess p-mTOR protein level as described treatments. **(K)** FCM determined the proportion of M2a macrophages (CD11b^+^CD206^+^) as described treatments. **(L)** WB analysis was used to assess p-mTOR and CD206 protein levels of macrophages as treatments with CC cell-derived exosomes. **(M)** FCM analysis of the M2a macrophage marker CD206 in IL-4-pretreated macrophages following CMTM6 knockdown. **(N)** WB examined the p-mTOR protein level of macrophages (IL-4 pretreated) when CMTM6 knockdown. For WB, the representative images were shown, n=3 independent experiments per group. For **(G, K, M)** data are presented as the mean ± SD, unpaired two-sided Student’s t-test, n=3 independent experiments per group.

Subsequently, we expounded which phenotype of macrophages the CMTM6 molecule is associated with. Through the analysis of online databases, we identified a negative correlation between CMTM6 expression and M1 macrophage (**Figure 4A**). In contrast, M2 macrophages are now recognized to have four phenotypes (M2a, M2b, M2c, M2d), and we found that CMTM6 is positively correlated with CD206, a classical marker of M2a (**Figure 4B**). Enrichment analysis of 50 genes positively correlated with CMTM6 expression in TCGA-CESC cohort showed that CMTM6 might play a role in cytosolic transport, which was confirm by GEPIA database (**Figure 4C**; **Supplementary Figures S4A, B**), suggesting that CMTM6 may be packaged in exosomes. Therefore, we investigated the potential presence of CMTM6 in exosomes, after characterizing the exosomes via TEM and NTA, WB analysis revealed significantly higher CMTM6 protein expression in exosomes compared to whole cell lysates (WCL) (**Figure 4D**; **Supplementary Figures S4C, D**). Consistently, cellular changes in CMTM6 also impact its levels in exosomes. Notably, when CC cells are treated with exosomal pharmacological inhibitors (GW4869), the CMTM6 expression in exosomes is altered, while its cellular levels remain unaffected (**Supplementary Figures S4E–G**). This suggests the presence of a compensatory intracellular degradation mechanism, likely mediated by both the proteasome and autophagy pathways, which prevents accumulation of CMTM6 upon inhibition of its exosomal secretion. The effect of GW4869 on exosomal CMTM6 mainly relies on these compensatory degradation processes rather than intracellular accumulation, which still needs further investigation to fully elucidate the underlying molecular mechanisms.

Recent studies have highlighted the crucial role of exosomes in regulating the interaction between tumor cells and TAMs (14, 16). We hypothesize that CMTM6 is transferred to TAMs via exosomes, promoting their differentiation into M2a. Confocal and WB analyses confirmed that macrophages can internalize exosomes (**Figures 4E, F**). Macrophages educated with CMTM6-altered exosomes (Exos^CMTM6 KO^ and Exos^TC1-sh-CMTM6^) had fewer M2a macrophages compared to those educated with control (WT-Exos and Exos^TC1-sh-nc^) (**Figures 4G, H**). To rule out the possibility that other changes in exosomal content caused this effect, we measured mRNA levels of several M2-related factors (TGF-β, IL-10, ARG1) in exosomes from CMTM6-knockdown cells, and found no significant differences compared to controls. This suggests the effect is mainly due to CMTM6 loss (**Supplementary Figure S5**). Due to the fact that macrophage polarization is usually accompanied by activation of intracellular signaling pathways, we firstly conducted gene set enrichment analysis (GSEA) found that CMTM6 in the TCGA-CESC cohort has a regulatory effect on mTOR signaling (**Figure 4I**). To support this, we further showed that CMTM6 knockdown in HeLa and Caski cells led to a significant decrease in phosphorylated mTOR levels, suggesting that CMTM6 may help sustain mTOR pathway activation in CC cells (**Supplementary Figure S6**). Building on this observation, we performed WB and found that macrophages (THP-1 and RAW264.7) treated with exosomes derived from CC cells exhibited activation of the mTOR signaling pathway. However, this phenomenon was attenuated when CMTM6 was absent in the exosomes (**Figure 4J**). Furthermore, mTOR inhibition also reversed the exosome-induced M2a macrophage polarization (**Figures 4K, L**). To further validate the regulatory role of CMTM6 in M2a polarization, we transfected IL-4-treated macrophages with CMTM6 siRNA. As expected, CMTM6 knockdown in macrophages reduced their tendency toward M2a activation, accompanied by decreased phosphorylation of the mTOR (p-mTOR) pathway (**Figures 4M, N**). These outcomes imply that CMTM6 was encapsulate into exosomes exerts an influence on M2a macrophage polarization by modulating the mTOR pathway.

### 3.5 M2a Macrophages educated by exosomal CMTM6 promote development of CC

**Figure 5:**
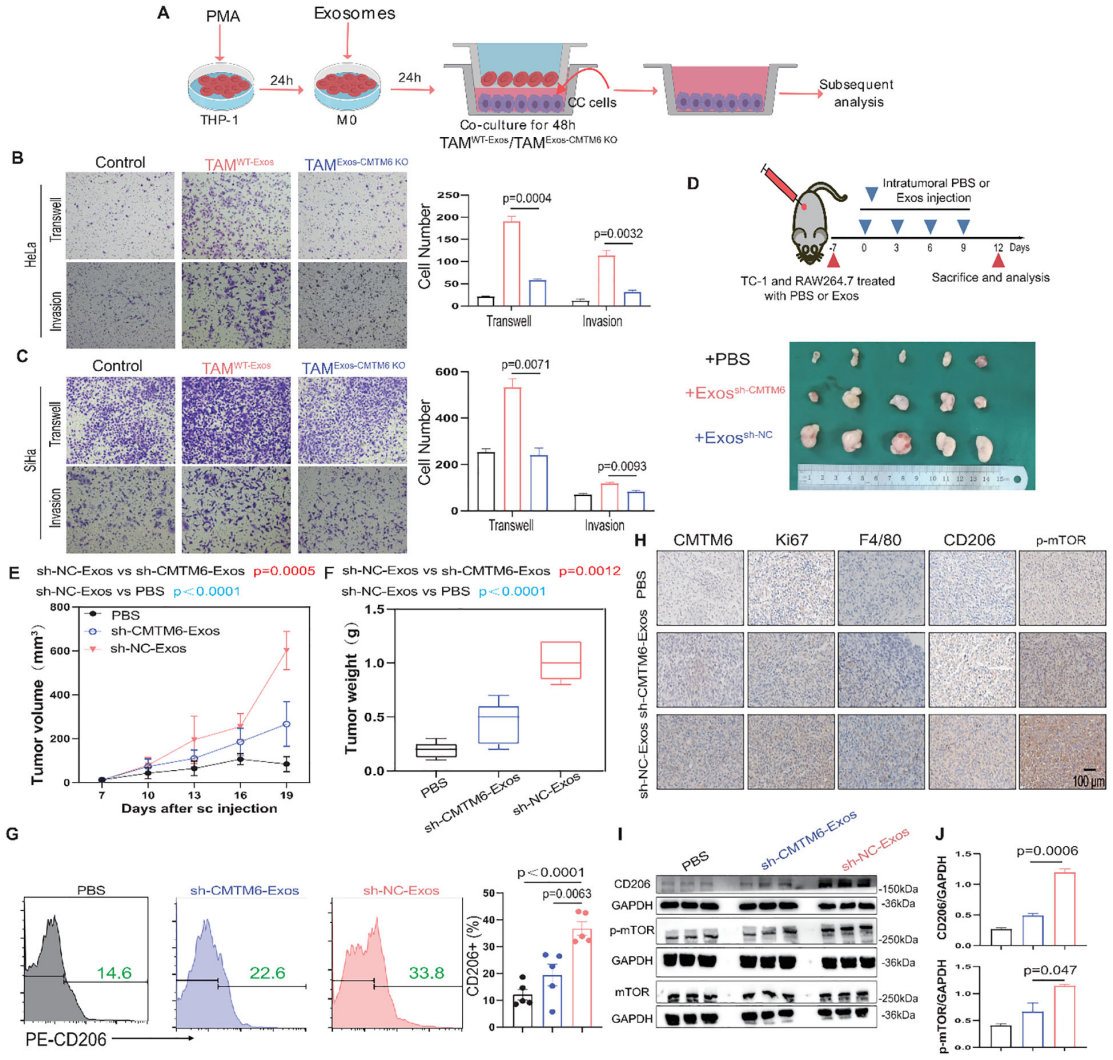
Exosomal CMTM6 educates M2a to promote CC progression. **(A)** THP-1 cells were treated with PMA, followed by the treatment of exosomes derived from tumor cells as indicated treatments to obtain TAMs. Then tumor cells cocultured with these TAMs were used to subsequent experiments. **(B, C)** The migration and invasion of HeLa and SiHa cells as cocultured with macrophages *in vitro* were evaluated using Transwell assays, respectively. Scale bar, 100μm. **(D)** Schematic illustration of *in vivo* validation of exosomal CMTM6 driving macrophage M2a and the exhibition of dissected tumors in different treatment groups. **(E, F)** The tumor volume **(E)** and weight **(F)** of tumor-bearing mice were compared after PBS, sh-NC-Exos and TC-1/sh-CMTM6-Exos treatment. Tumor volume was measured every three days. **(G)** The proportion of M2a macrophages (F4/80^+^CD206^+^) in each group (PBS, sh-NC-Exos and TC-1/sh-CMTM6-Exos) was detected by FCM. **(H)** Representative IHC staining images of CMTM6, Ki67, F4/80, CD206, p-mTOR expression in mice CC tissues of three groups. Scale bar, 100μm. **(I)** WB and quantification analysis was used to assess CD206 and p-mTOR protein levels as described treatments. For **(B, C)** data are presented as the mean ± SD, unpaired two-sided Student’s t-test, n=3 independent experiments per group. For **(E, G)** data are presented as the mean ± SEM, one-way ANOVA followed by Tukey multiple comparison test, n=5 per group. For **(J)**, data are presented as the mean ± SD, n=3, unpaired two-sided Student’s t-test.

M2a is a malignant macrophage phenotype known to foster tumor progression (17, 18). We constructed a coculture system to elucidate the function of tumor exosomal CMTM6 internalized by TAMs (TAMs/WT−Exos or TAMs/Exos-CMTM6 KO). Subsequently, tumor exosome-induced TAMs were cocultured with tumor cells for 48 h, and then these tumor cells were collected for Transwell assays (**Figure 5A**). As expected, the migrated and invasive ability of CC cells were notably increased after coculturing with exosome-pulsed TAMs, especially coculturing with TAMs^WT−Exos^ (**Figures 5B, C**). To validate the above results *in vitro*, we conducted *in vivo* experiments. We first verified whether exosomes alone can directly promote tumor growth *in vivo*, TC–1 cells were implanted subcutaneously into C57BL/6 mice, followed by three intratumoural injections of TC–1–derived exosomes or PBS without macrophage supplementation. Tumor volumes did not differ significantly between the exosome–treated and control groups (**Supplementary Figure S7**), indicating that exosomes by themselves are insufficient to drive tumor outgrowth in the absence of macrophages. We then validated the macrophage−dependent effect *in vivo*. TC-1 and RAW264.7 cells, pretreated with PBS or exosomes, were mixed and subcutaneously injected into the right abdomen of mice. Subsequently, PBS or exosomes from TC-1/sh-NC or TC-1/sh-CMTM6 cells were intratumorally injected into the tumor-bearing mice. Notably, the sh-NC-Exos group displayed larger tumors with faster growth rates compared to other (**Figures 5D–F**). In addition, FCM showed more m2a macrophage infiltration in tumor tissue of sh-NC-Exos group (**Figure 5G**). IHC analysis further confirmed that exosomal CMTM6 accelerates CC progression and induces a higher proportion of M2a, accompanied by the activation of the mTOR pathway (**Figure 5H**). Similar findings were obtained in WB and quantification analysis (**Figures 5I, J**). Together, exosomal CMTM6 can drive the M2a macrophages and promote the progression of CC.

### 3.6 M2a exhibit increased cytokine/chemokine production mediated by exosomal CMTM6

**Figure 6:**
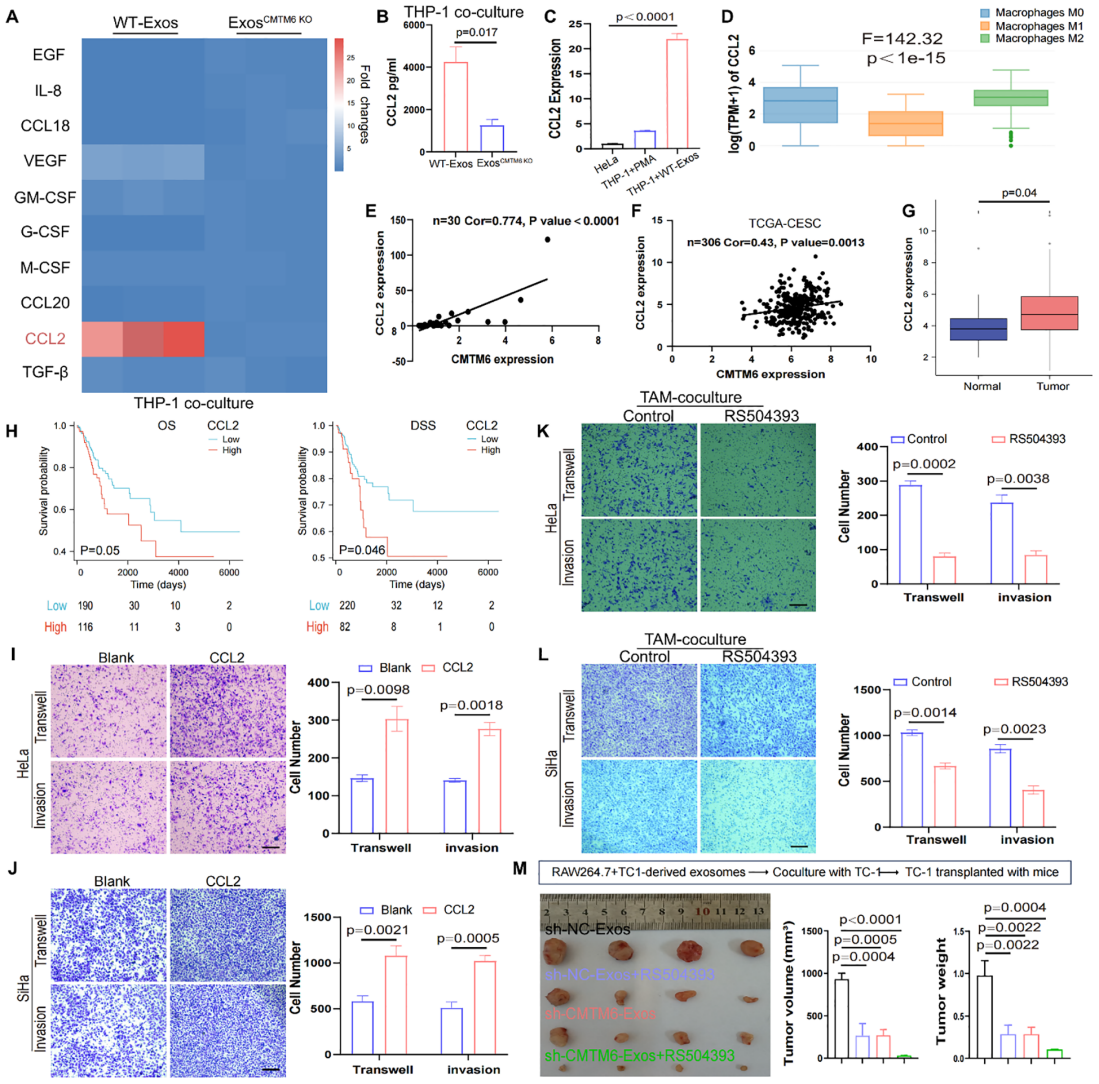
Exosomal CMTM6 promotes the secretion of CCL2 in macrophages. **(A)** The mRNA levels of 10 cytokines or chemokines in macrophages as treatment with WT−Exos and Exos-CMTM6 KO were quantified by qPCR. **(B)** The concentrations of CCL2 in the supernatant of THP-1-derived macrophages after exosomes treatment were measured by ELISA. **(C)** The CCL2 mRNA levels comparison of cells as indicated treatment. **(D)** mRNA Expression of CCL2 in M0, M1 and M2 macrophages using GEPIA database. **(E)** Correlation analysis for CMTM6 and CCL2 mRNA levels in CC samples. **(F)** The correlation between CMTM6 and CCL2 mRNA levels was analyzed by TCGA-CESC cohort. **(G)** The mRNA expression of CCL2 in normal and cancer tissues in the TCGA-CESC was analyzed. **(H)** The Kaplan-Meier survival curves was drawn to state the relationship between CCL2 mRNA expression and OS (left) and DSS (right) in CC patients. Analyzed using the log-rank test. **(I, J)** The migration and invasion of HeLa and SiHa as treatment with control and CCL2 were demonstrated using Transwell assays. Scale bar, 100μm. **(K, L)** The migration and invasion of cocultured HeLa and SiHa as treatment with control and RS504393 were demonstrated using Transwell assays. Scale bar, 100μm. **(M)** RAW264.7 treated with exosomes from TC-1 cells and then coculture with TC-1. The co-cultured TC-1 cells were transplanted into mice and mice were treated with saline or RS504393 and subcutaneous tumors were isolated and tumor volume was recorded on 22th day. The images of excised tumors from each group at the experimental endpoint (left). Tumor volumes (middle) and tumor weights (right) at the endpoint were compared across the four groups. For **(B, C)** data are presented as the mean ± SD, unpaired two-sided Student’s t-test, n=3 independent experiments per group. For **(G)** data are presented as the mean ± SEM, Wilcoxon rank sum test. For **(H)**, statistical analysis is using log-rank test. For **(I–L)** data are presented as the mean ± SD, unpaired two-sided Student’s t-test, n=3 independent experiments per group. For **(M)** data are presented as the mean ± SEM, one-way ANOVA followed by Dunnett’s multiple comparison test, n = 4 per group.

Cytokine secretion, a key indicator of macrophage function, was investigated to uncover how exosomal CMTM6 enhances macrophage-driven migration and invasion of CC cells. Employing qPCR, we conducted a comprehensive analysis of the expression profiles of ten inflammatory mediators and chemokines implicated in mTOR pathway activation in macrophages. Intriguingly, the data unveiled a marked elevation of CCL2 expression in macrophages stimulated with normal exosomes (**Figure 6A**). ELISA results further confirmed the increased secretion of CCL2 (**Figure 6B**). Simultaneously, the CCL2 baseline level in macrophages greatly surpassed that found in HeLa cells, while co-culturing exosomes with macrophages notably enhanced the CCL2 expression of macrophages (**Figure 6C**). Moreover, GEPIA database indicated higher CCL2 expression in M2 compared to M1 and M0 macrophages and a strong correlation between CMTM6 and CCL2 expression was confirmed by our cohort data and TCGA datasets (**Figures 6D–F**). Thus, we suggest that CMTM6 enhances CCL2 secretion by driving M2a polarization. As expected, CCL2 was highly expressed in CC tissues, linked to poor prognosis (**Figures 6G, H**). Next, we sought to determine whether CCL2 levels contribute to tumorigenic processes in CC. Using Transwell assays, we first verified that CCL2 markedly enhanced the migratory and invasive potential of CC cells (**Figures 6I, J**). Finally, we explored whether the elevated CCL2 contribute to the tumor-promoting effects mediated by M2a macrophages induced by CC-derived exosomal CMTM6. To address this, CC cells co-cultured with exosome-treated macrophages were subjected to RS504393 (CCL2 receptor antagonist) intervention. Transwell assays revealed that RS504393 effectively suppressed tumor cell migration and invasion (**Figures 6K, L**). To further evaluate this regulatory axis *in vivo* aiming to dissect the individual and combined effects of CMTM6 depletion and CCL2 inhibition. Tumor-bearing mice treated with sh-CMTM6-Exos or RS504383 alone exhibited moderate but comparable reductions in tumor growth, indicating that both interventions can restrain tumor progression to a certain extent. Notably, the combination of sh-CMTM6-Exos and RS504383 resulted in the most significant tumor suppression (**Figure 6M**), suggesting a potential additive or synergistic interaction between exosomal CMTM6 and CCL2 signaling in promoting CC progression. Together, these findings strongly suggest that macrophage-derived CCL2 may be a key cytokine mediating the development of CC. In summary, CMTM6-mediated exosomes can promote the polarization of M2a. These macrophages then release CCL2 to increase tumorigenicity.

### 3.7 Clinical significance of the CMTM6/M2a polarization/CCL2 axis in CC

**Figure 7:**
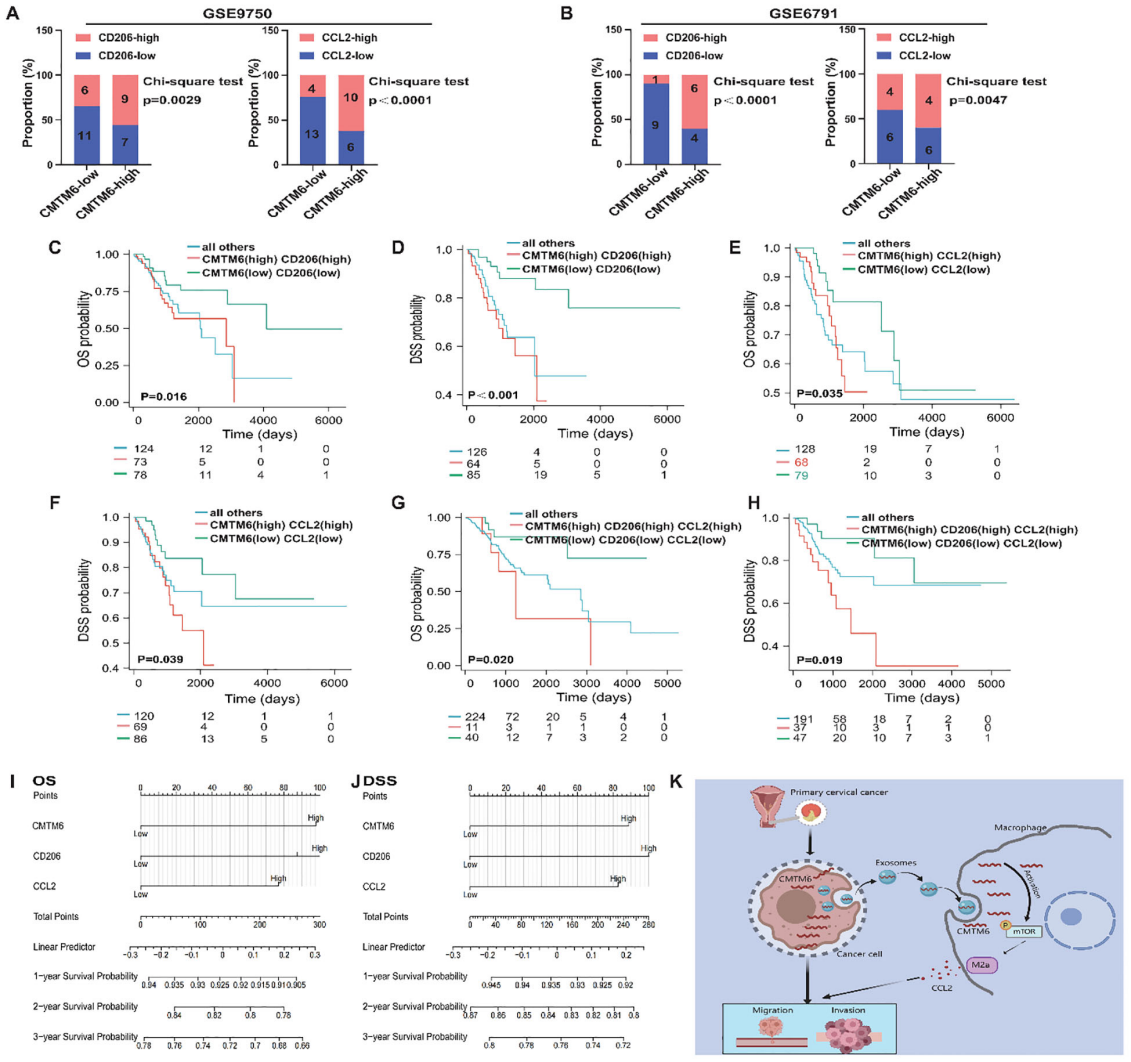
The CMTM6/M2a/CCL2 axis represents a key indicator for prognosis in CC patients. **(A, B)** Proportion of CD206 (left) and CCL2(right) mRNA expression levels in tumor tissues with low or high CMTM6 expression in GSE9750 **(A)** and GSE6791 **(B)**. **(C, D)** Kaplan-Meier analysis was used to determine the prognostic value of OS and DSS in CC patients with CMTM6highCD206high and CMTM6lowCD206low. **(E, F)** Kaplan-Meier analysis was conducted to assess the prognostic value of OS and DSS in CC patients with CMTM6highCCL2high and CMTM6lowCCL2low mRNA expression profiles. **(G, H)** Kaplan–Meier analysis to determine the prognostic values of CMTM6/M2a/CCL2 axis for patient OS and DSS. **(I, J)** A nomogram was constructed based on the independent prognostic factors of OS and DSS. **(K)** A depiction of how exosomal CMTM6 facilitates communication between tumor cells and macrophages, driving the formation of M2a in CC (created with MedPeer). This highlights the potential of exosomal CMTM6 as a promising diagnostic biomarker and therapeutic target for CC immunotherapy. For **(A, B)** statistical analysis was performed using the chi-square test. For **(C–H)** the survival analysis was analyzed by log rank test.

To thoroughly investigate the clinical significance of the CMTM6/M2a polarization/CCL2 axis, we performed an extensive evaluation of CMTM6 expression in CC specimens and analyzed its relationship with the corresponding levels of CD206 and CCL2. Data derived from the GEO databases (GSE9750, GSE6791) revealed a strong correlation between elevated CMTM6 mRNA expression and increased M2a macrophage infiltration (CD206) as well as higher CCL2 mRNA levels, and this trend also existed in samples with low CMTM6 expression (**Figures 7A, B**). Furthermore, survival analyses based on TCGA-CESC indicated that CC patients exhibiting higher mRNA expression levels of CMTM6, CD206, or CCL2 had markedly worse OS and DSS outcomes compared to their counterparts with lower expression levels (**Figures 7C–F**). These observations highlight the substantial clinical relevance of CMTM6, CCL2, and M2a macrophage infiltration as powerful prognostic biomarkers in CC. Specifically, patients characterized by elevated CMTM6 and CCL2 mRNA expression, coupled with a high proportion of CD206-positive macrophages, demonstrated the most unfavorable OS and DSS outcomes (**Figures 7G, H**). Building on these prognostic indicators, we constructed a detailed nomogram to quantitatively predict the OS and DSS of CC patients with high accuracy (**Figures 7I, J**). In summary, our findings shed light on the prognostic significance of CMTM6/M2a polarization/CCL2 axis in CC. Additionally, **Figure 7K** presents a comprehensive and systematic overview of the pathways, processes, and mechanisms explored in this study.

## 4 Discussion

According to previous reports, tumor development is influenced by non-malignant cells in the TME (19). Among these non-malignant cells, macrophages have been identified to play a crucial role in the TME, promoting tumor progression by facilitating cancer cell migration and invasion while suppressing anti-tumor immunity (20). Macrophages can be divided into M1 and M2 types, with varying functions in the TME. M1 macrophages typically possess tumor suppressive functions, whereas M2 macrophages promote tumor development (4, 21). The findings of this study reveal a critical role of CMTM6 in the tumor microenvironment. The absence of CMTM6 significantly hinders the recruitment and infiltration of TAMs, thereby suppressing tumor development and progression. This highlights CMTM6 as a key regulator of the tumor microenvironment, particularly in modulating immune cell dynamics. Furthermore, CMTM6 was shown to drive the formation of the M2a malignant macrophage phenotype, this dual role suggests that CMTM6 promotes oncogenesis by both facilitating TAM recruitment and reprogramming macrophages, making it a promising target for cancer therapy.

In addition to its macrophage-dependent effects, our data also suggest that CMTM6 exerts tumor-intrinsic functions. CMTM6 knockdown directly suppressed the proliferation, migration, invasion, and survival of CC cells, indicating a cell-autonomous oncogenic role. Notably, tumor growth inhibition was observed even in the absence of macrophages, further supporting the involvement of macrophage-independent mechanisms. Moreover, beyond macrophages, CMTM6 may modulate the activity of other immune cells within the TME. Although not directly assessed in this study, prior evidence has implicated CMTM6 in stabilizing PD-L1 expression, thereby impacting T cell–mediated immune responses. The potential regulation of dendritic cells, myeloid-derived suppressor cells, or T lymphocytes by CMTM6 could contribute to its immunomodulatory functions and warrants further investigation (20, 22, 23).

There is increasing evidence that tumor-derived exosomes migrate to macrophages and induce their polarization into the M2 type (24). Although previous studies have reported the overexpression of CMTM6 in breast cancer, liver cancer, and head and neck squamous cell carcinoma, its specific function and molecular mechanism in promoting CC remain unclear (5, 25, 26). Our results revealed that CMTM6 is highly expressed in CC and significantly associated with poor prognosis in patients. *In vitro* and *in vivo* experiments confirmed that CMTM6 expression benefits the biological characteristics of tumor cells. Crucially, exosomes derived from CC cells contain abundant CMTM6 and can be internalized by macrophages, resulting in the M2a macrophages polarization.

Given the genetic alterations in tumor cells and the limited success of existing treatments, recent research has increasingly shifted toward understanding and targeting the TME as a promising therapeutic avenue (27). Notably, exosomes have emerged as critical mediators within the TME, facilitating intercellular communication by transferring bioactive molecules to recipient cells. For instance, Exosomal lncARSR promotes M2 macrophage polarization via STAT3 activation, advancing renal cell carcinoma (28). Likewise, exosomal CD73 from head and neck squamous cell carcinoma induces M2 polarization, reprogramming the TME (6). This study uncovered a novel mechanism by which CMTM6, encapsulated in CC-derived exosomes, is transferred to macrophages, thereby activating mTOR signaling and driving their polarization toward the pro-tumorigenic M2a. Importantly, pharmacological inhibition of mTOR signaling was able to reverse this process, highlighting the therapeutic potential of targeting this pathway. The validity of these observations was further reinforced by *in vivo* experiments, which provided strong evidence supporting the role of exosomal CMTM6 in modulating macrophage behavior within the TME.

The M2a macrophages is instrumental in orchestrating tumor growth, migration, and angiogenesis by secreting key growth factors and cytokines (29–31). CCL2 is a key chemokine involved in cancer development, with signaling pathway significantly contributing to the progression of various cancers (32) The CCL2 drives tumor growth through the PI3K/AKT/mTOR pathway, which is associated with poor prognosis in breast cancer (33). Additionally, the P38-MAPK pathway enhances the growth and invasion of ovarian cancer cells (14). In our study, we demonstrated that M2a-type of macrophages induced by CC cell-derived exosome CMTM6 establishes sustained interaction between tumor cells and macrophages via the CCL2 secretion, creating an inflammatory microenvironment that promotes CC development, and blocking CCL2 signaling can suppress the tumor-promoting effect of M2a macrophages. Our study elucidates the molecular mechanisms underlying CC development, highlighting the mutual regulation of multiple molecules and gene regulation.

In summary, we elucidated a dual mechanism by which CMTM6 facilitates CC progression. First, the elevated expression of CMTM6 in tumor cells promotes the proliferation, migration, and invasion of CC. Second, exosomal CMTM6 secreted by CC cells activates the mTOR pathway, leading to the polarization of M2a macrophages. Further investigations revealed that M2a macrophages polarized by CMTM6 enhance CC progression by secreting CCL2. Our findings not only underscore the clinical significance of the CMTM6-M2a-CCL2 axis in CC but also propose exosomal CMTM6 as a promising novel target for immunotherapy. These insights not only deepen our understanding of the tumor-macrophage interaction but also open new avenues for the prevention and treatment of CC. CMTM6 holds potential as both a biomarker for assessing the future risk of CC and a therapeutic target in cancer treatment.

## Data Availability

The datasets presented in this study can be found in online repositories. The names of the repository/repositories and accession number(s) can be found in the article/**Supplementary Material**.
