# Supplementary material for: Oncogenic CMTM6 drives M2a macrophages formation and fuels cervical cancer progression
Source: Front Immunol. 2025 Jul 21;16:1621816. doi: 10.3389/fimmu.2025.1621816 (PMC12319053; doi:10.3389/fimmu.2025.1621816)
Supplement: Supplementary file 1 [file DataSheet1.docx]

Supplementary Material

Oncogenic CMTM6 Drives M2a Macrophages Formation and Fuels Cervical Cancer Progression

Bo Yin, Chun Chen, Baoyou Huang，Jianyi Ding, Haoran Hu, Huijuan Zhou, Yashi Zhu, Tiefeng Huang, Xiang He*, Yuan Lu*, Lingfei Han*

* Corresponding Author:

Lingfei Han, E-mail: lingfeihan@tongji.edu.cn. Yuan Lu, E-mail: yuanlu@fudan.edu.cn. Xiang He，E-mail: heroxiang@hotmail.com.

These authors contributed equally: Bo Yin, Chun Chen, Baoyou Huang

This file includes: Original gels; Supplementary Table S1- S3; Supplementary Figure S1-S7 and Figure Legends.


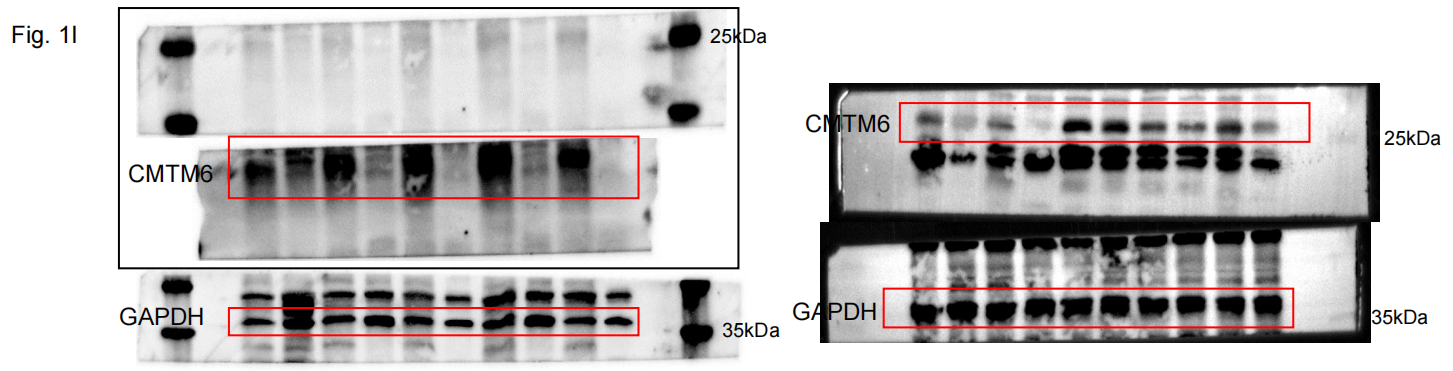


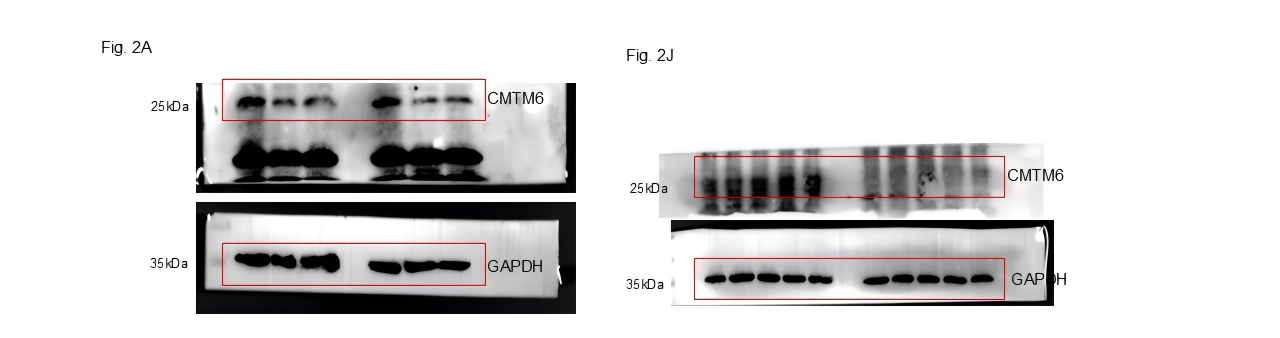


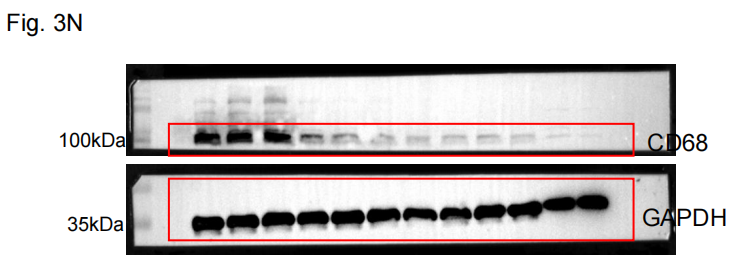


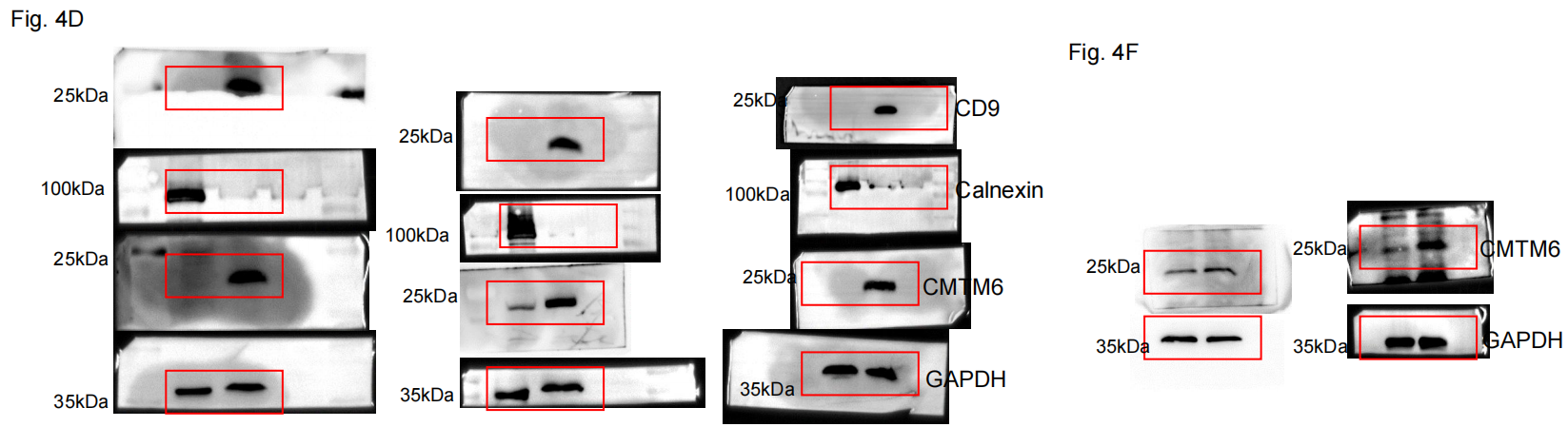


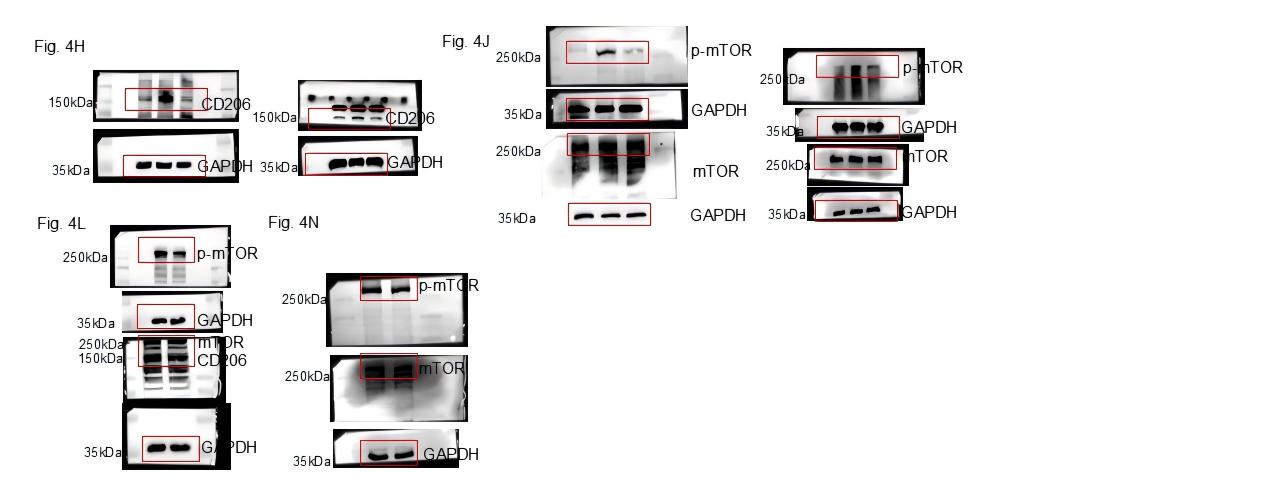


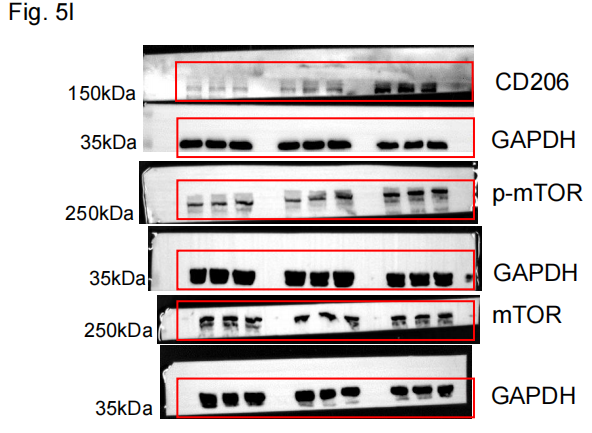


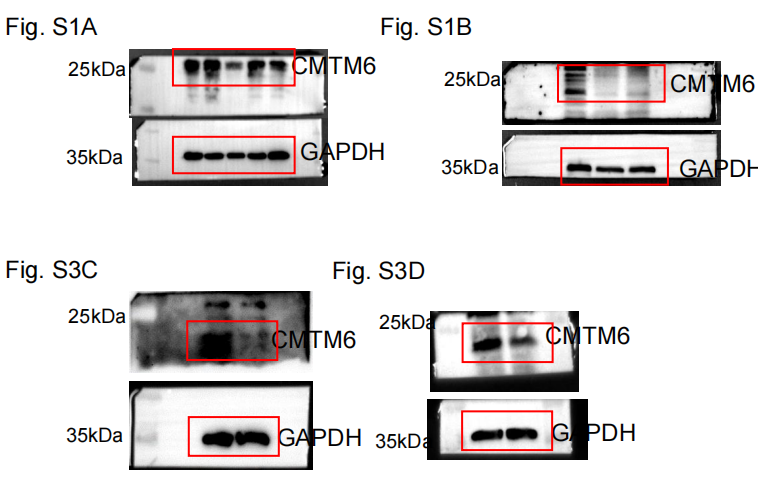


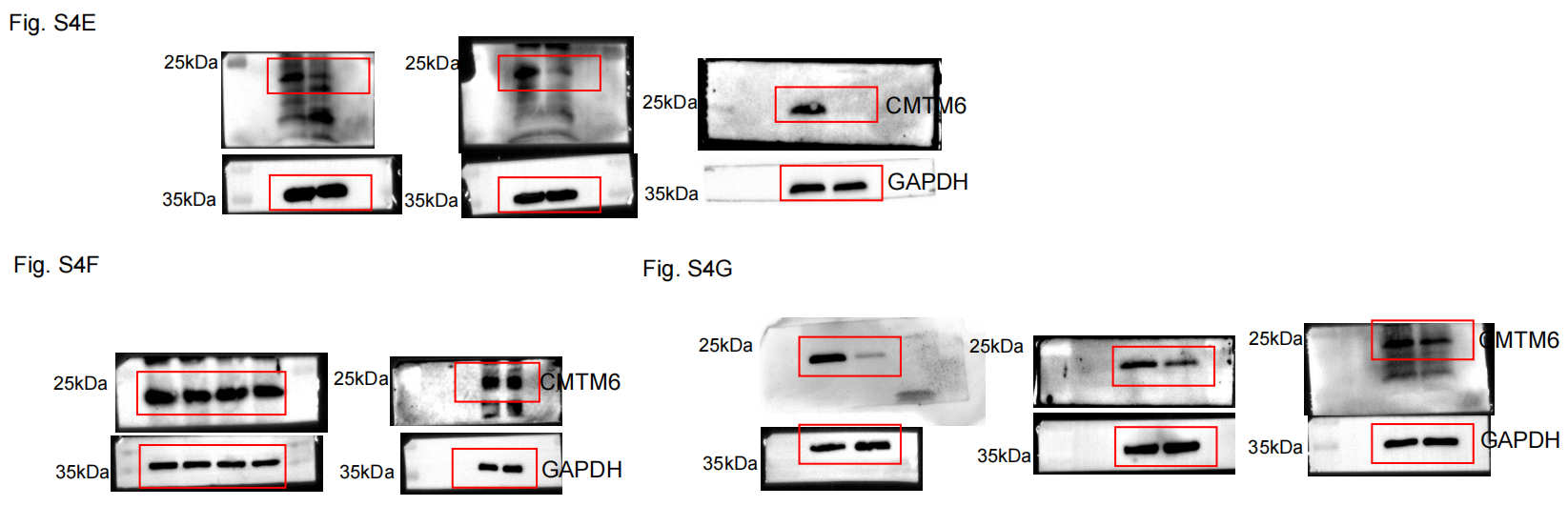


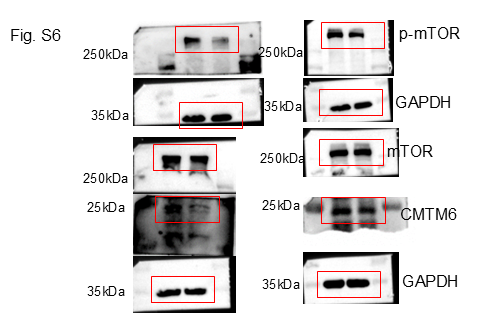


**Table S1. The forward and reverse primer sequence were listed**

| Gene | Species | FORWARD (5’ to 3’) | REVERSE (5’ to 3’) |
| --- | --- | --- | --- |
| GAPDH | Human | GAAGGTGAAGGTCGGAGTC | GAAGATGGTGATGGGATTTC |
| CMTM6 | Human | TTTCCACACATGACAGGACTTC | GGCTTCAGCCCTAGTGGTAT |
| CCL2 | Human | AGCAGCAAGTGTCCCAAAGA | TTGGGTTTGCTTGTCCAGGT |
| IL-10 | Human | CTGAGAACCAAGACCCAGACA | AAAGGCATTCTTCACCTGCTCC |
| TGFβ | Human | CACTCTCAAACCTTTACGAGACC | CGTTGCTAGGGGCGAAGATG |
| ARG1 | Human | TGACGGACTGGACCCATCTT | GGCTTGTGATTACCCTCCCG |

**Table S2: Antibodies and Reagents**

| **Name** | **Catalog** | **Company** | **Application** |
| --- | --- | --- | --- |
| GAPDH | AB2100 | NCM Biotech | 1:20000(WB) |
| CMTM6 | 55829 | Cell Signaling | 1:1000(WB) |
| CMTM6 | 860396 | ZENBIO | 1:100(IHC) |
| Ki67 | A2094 | ABclonal | 1:200(IHC) |
| CD68 | Orb197999 | Biorbyt | 1:200(IHC); 1:1000(WB) |
| F4/80 | A23788 | ABclonal | 1:100(IHC) |
| CD9 | EXOAB-CD9A-1 | System Bioscience | 1:1000(WB) |
| Calnexin | 2433 | Cell Signaling | WB (1:1000) |
| CD206 | sc-376232 | Santa Cruz | 1:100(IHC); 1:1000(WB) |
| p-mTOR(S2448) | T56571 | Abmart | 1:1000(WB); 1:100(IHC) |
| mTOR | T55306 | Abmart | 1:1000(WB) |
| APC anti-mouse/human CD11b | 101212 | Biolegend | FCM |
| Brilliant Violet 421™ anti-human CD274 (B7-H1, PD-L1) | 329714 | Biolegend | FCM |
| PE anti-human CD47 | 323108 | Biolegend | FCM |
| FITC anti-human CD11b | 982614 | Biolegend | FCM |
| APC anti-human CD206/MMR | 321110 | Biolegend | FCM |
| PerCP/Cyanine5.5 anti-mouse F4/80 | E-AB-F0995J | Elabscience | FCM |
| PE Anti-mouse CD206/MMR | E-AB-F1135D | Elabscience | FCM |

**WB: western blotting; IHC: immunohistochemistry; FCM: flow cytometry.**

| **Reagents** | **Catalog** | **Company** |
| --- | --- | --- |
| BD Horizon™ CFSE | 565082 | BD Biosciences |
| InVivoMAb anti-mouse F4/80 | BE0206 | BioXCell |
| CellTracker™ CM-Dil | C7001 | Invitrogen |
| Sapanisertib | HY13328 | MedChemExpress |
| RS504393 | HY15418 | MedChemExpress |

**Table S3: The sequences used in siRNA, shRNA work and CRISPR/Cas9 knockout system**

| FORWARD (5’ to 3’) | Application |
| --- | --- |
| GAAGUUGUAUCACAAUGUATT | siRNA-1 |
| GAGAGAGUUGAUACCACAATT | siRNA-2 |
| GGCCTTCATCTGTGAAGAGGT | shRNA-1 |
| GCTGAAATTGCTGCAATTGTG | shRNA-2 |
| TCACAATGTACTTTATGTGG | CRISPR/Cas9 |


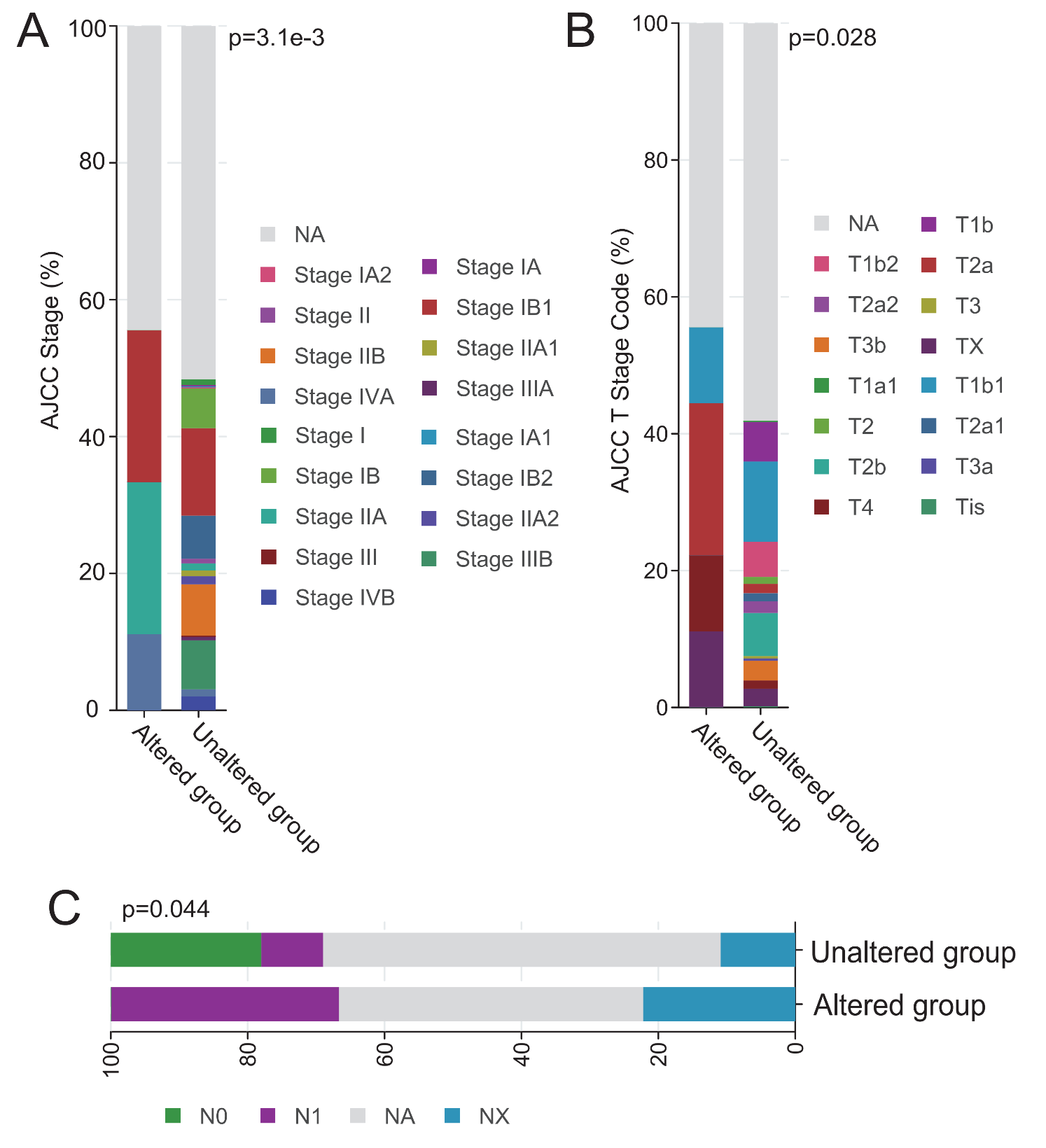


Fig. S1 The CNV of CMTM6 are associated with poor prognosis.

(A) The CMTM6 altered group is associated with an advanced AJCC stage (cbioportal).

(B) The CMTM6-altered group is correlated with a more advanced AJCC T stage (cbioportal).

(C) CMTM6 genetic alterations are associated with lymph node metastasis in patients (cbioportal).


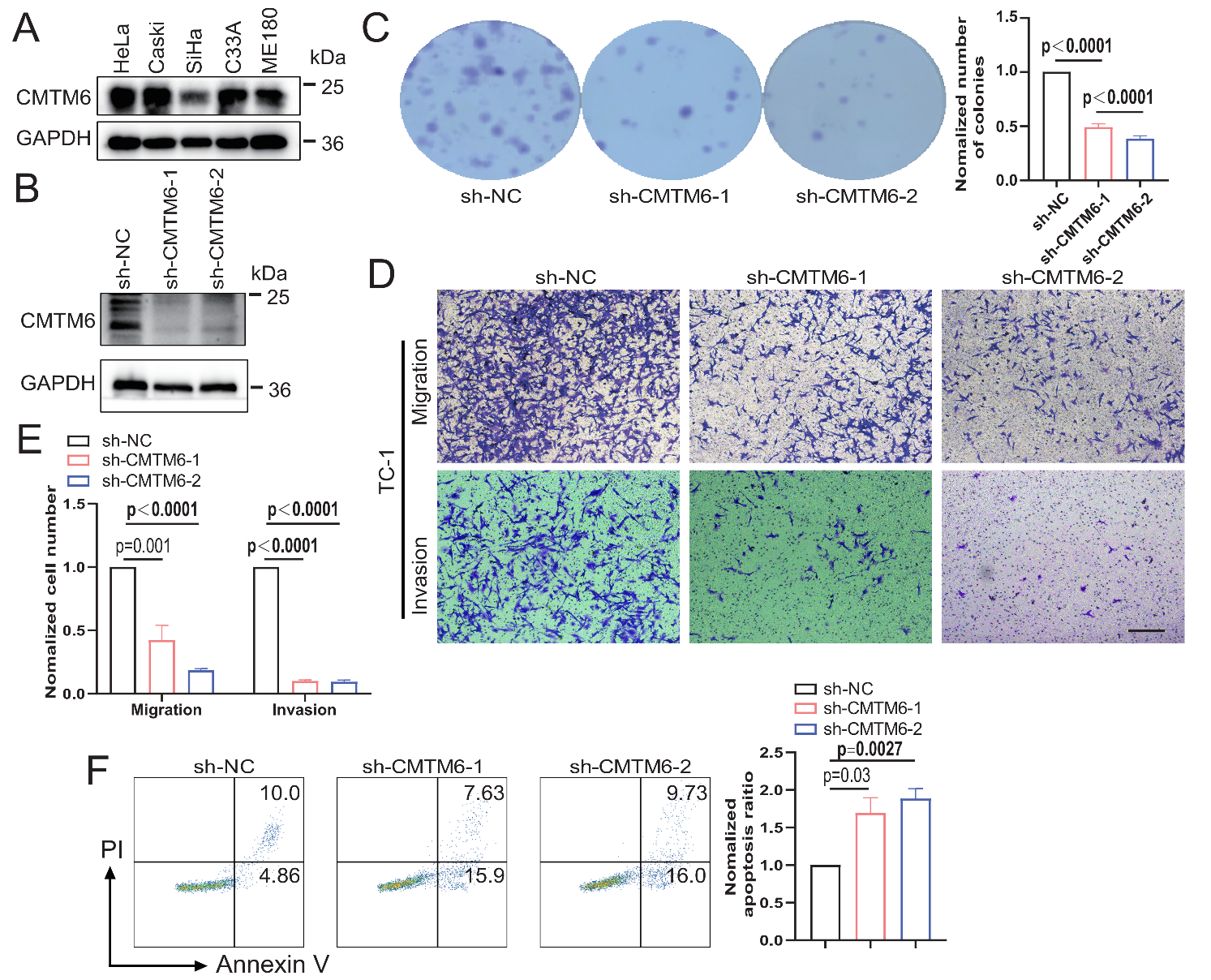


**Fig. S2** The change of CMTM6 expression affects the biological function of CC.

(A) WB was performed to detected CMTM6 expression in HeLa, Caski, SiHa, C33A and ME180 cell lines.

(B) WB showed that the knockdown efficiency of CMTM6 shRNA on TC-1 cells.

(C) The proliferation of TC-1 cells as indicated treatments were evaluated.

(D) The migration and invasion of TC-1 cells according to the treatments applied were demonstrated using transwell assays.

(E) Quantification of the number of migrating and invading TC-1 cells under different treatments.

(F) The apoptosis of TC-1 cells as described treatments was studied by flow cytometry.

For C, E, F, the representative results were shown, data are presented as the mean ± SD, unpaired two-sided Student's t-test, data are shown as normalized to the NC group which was set to 1 after normalization, n=3 independent experiments per group.


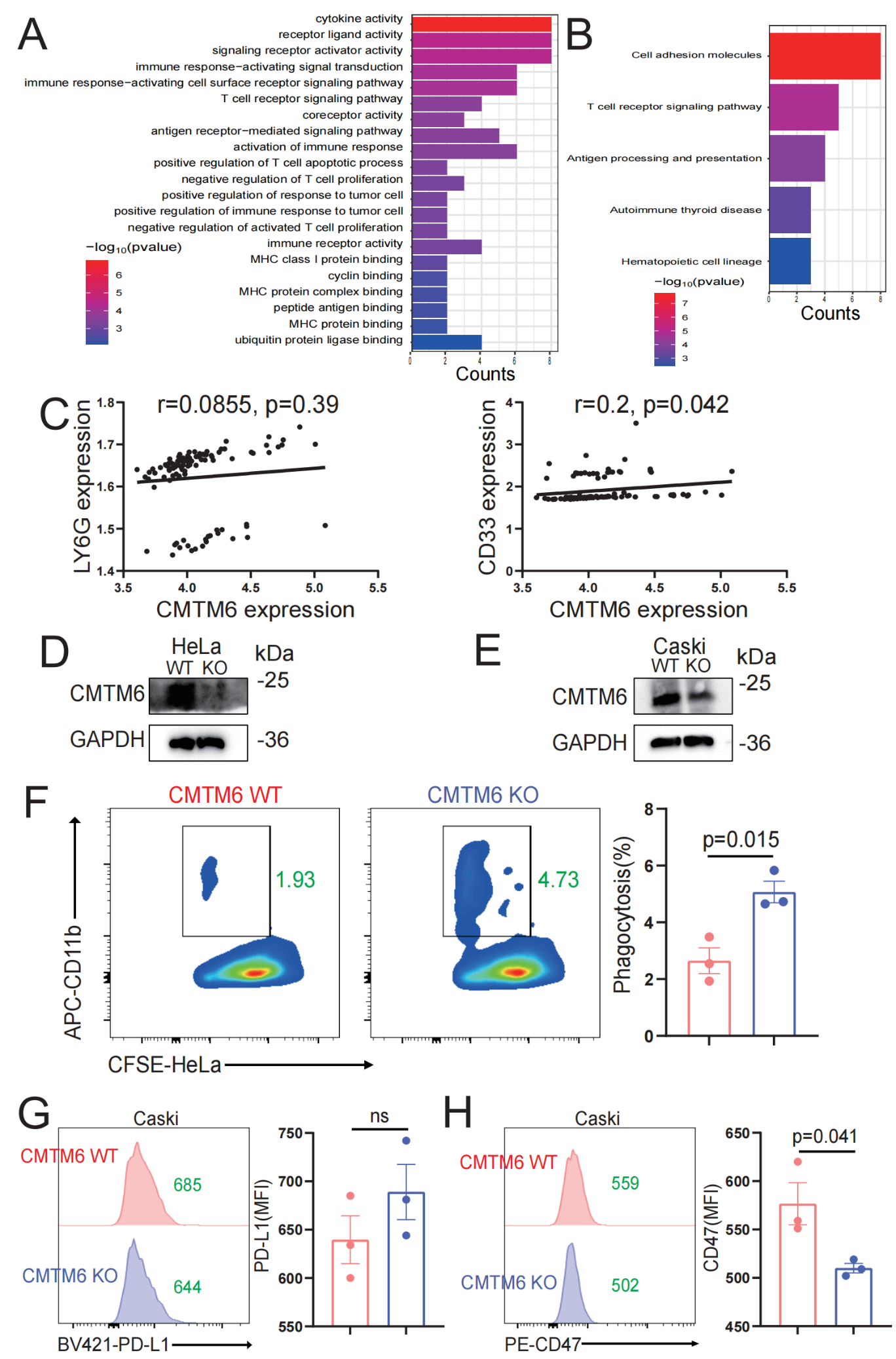


**Fig. S3** CMTM6-mediated CD47 expression in CC enables immune evasion by macrophages.

(A-B) GO and Kyoto Encyclopedia of Genes and Genomes (KEGG) analysis of interactors of CMTM6.

(C) Spearman Correlation analysis of LY6G^+^ neutrophils and CD33^+^ MDSCs cells and the CMTM6 mRNA expression in GSE63514 (n=104).

(D-E) WB showed that the CMTM6 sgRNA efficiency on HeLa and Caski.

(F) Flow cytometry was used to assess tumor cell phagocytosis by macrophages in each group.

(G-H) The expression of PD-L1 (F) and CD47 (G) in Caski cells as indicated group was detected by flow cytometry. The flow cytometry results were quantified using MFI values. For F, data are presented as the mean ± SD, unpaired two-sided Student's t-test, n=3 independent experiments per group. For G, H, data are presented as the mean ± SD, unpaired two-sided Student's t-test, n=3 independent experiments per group, the data are presented as the MFI values.


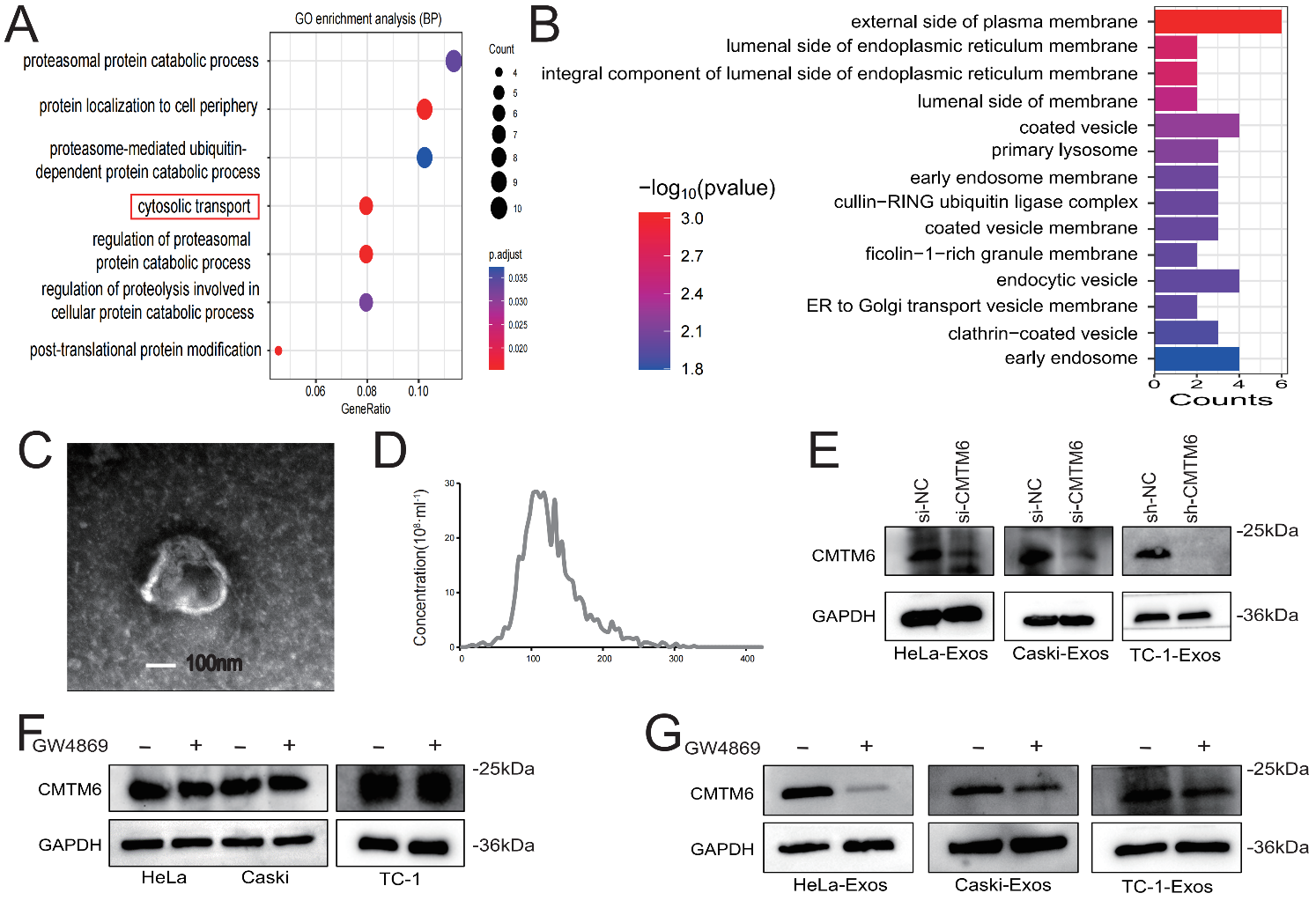


**Fig. S4** CMTM6 protein is encapsulated in the exosomes of CC cells.

(A-B) GO analysis of genes related with CMTM6 via GEPIA and LinkedOmics database (https://www.linkedomics.org/).

(C-D) Phenotypic analysis of exosomes derived from HeLa cells was conducted using TEM (C) and NTA (D). Scale bar: 100 nm.

(E) The expression of CMTM6 in exosomes originated from the cell line with downregulated CMTM6 expression was assessed via WB.

(F) WB analysis of the expression of CMTM6 in cells as described treatments.

(G) WB analysis of CMTM6 expression in cell-derived exosomes as described treatments.


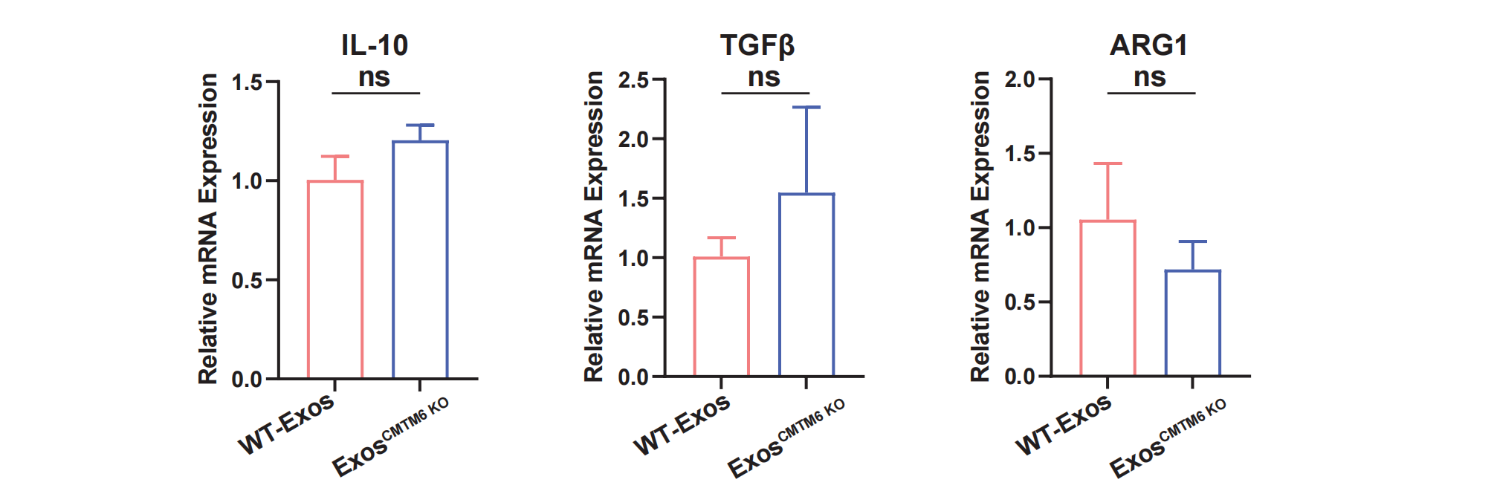


**Fig. S5** M2 markers unchanged by exosomal CMTM6 knockdown.

qPCR analysis of mRNA expression levels of M2-associated factors (IL-10, TGF-β, and ARG1) in exosomes derived from wild-type (WT) and CMTM6-knockout HeLa cervical cancer cells. ns, not significant.


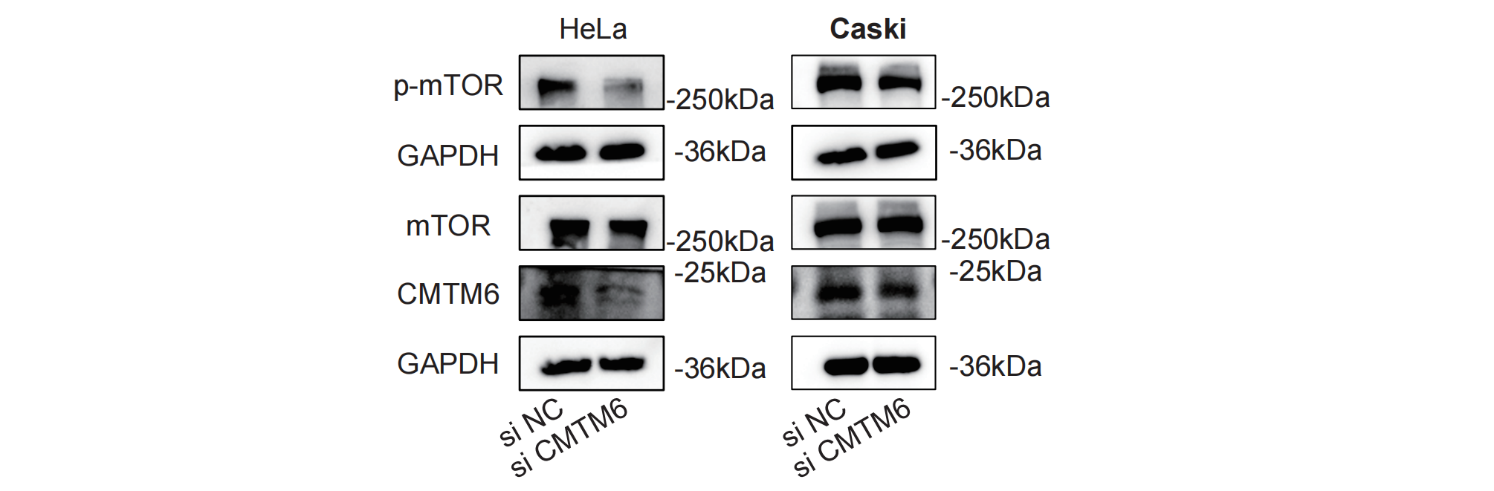


**Fig. S6 Knockdown of CMTM6 inhibits mTOR signaling activation in tumor cells**

WB was used to detect the expression level of p-mTOR after CMTM6 knockdown in tumor cells (HeLa and Caski). Representative WB images were displayed and n=3 independent experiments per group.

**
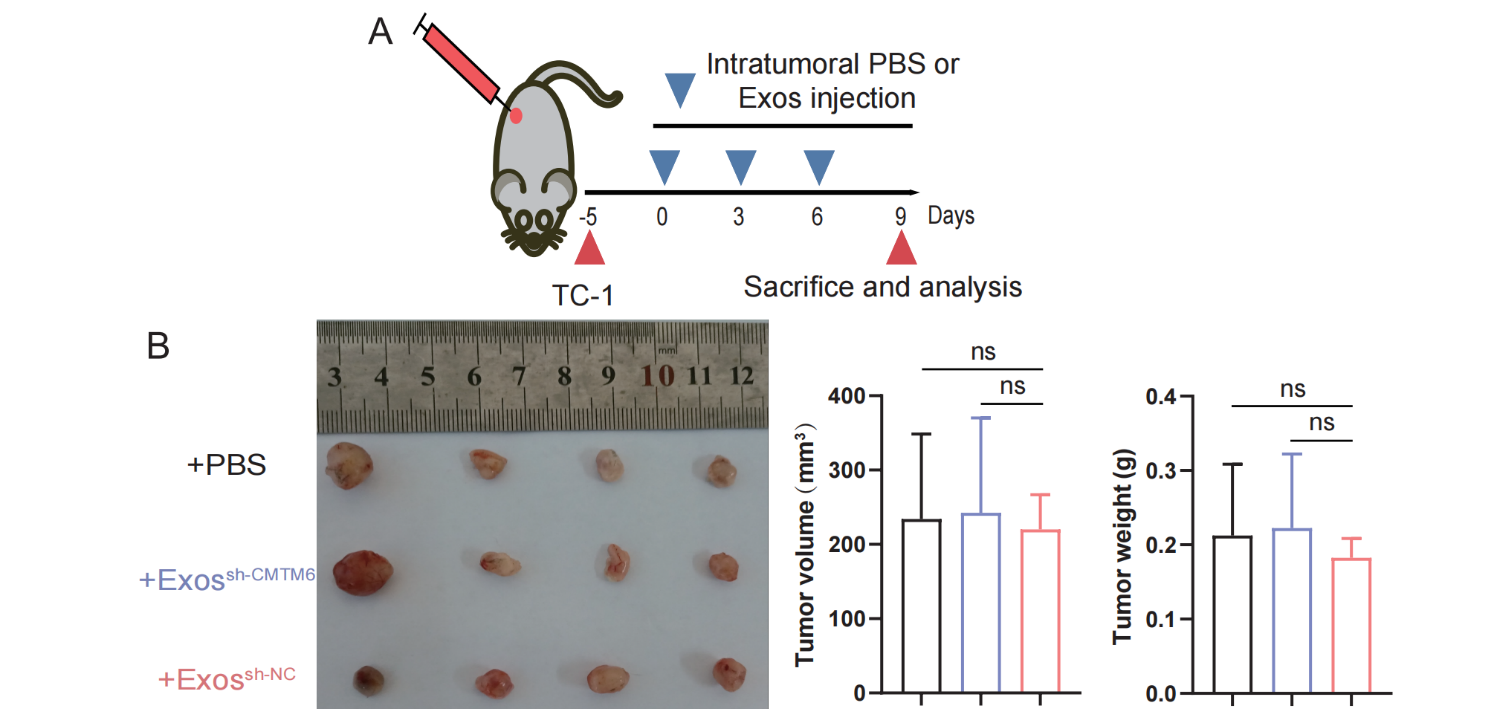

Fig. S7 Exosome injection alone has no significant effect on tumor growth.**

(A) TC-1 cells were subcutaneously implanted into C57BL/6 mice (n = 4 per group). Starting on day 5 post-implantation, mice received intratumoral injections of PBS, sh-NC-Exos, or sh-CMTM6-Exos (40 μg per dose) on days 0, 3, and 6 days. (B) The images of excised tumors from each group at the experimental endpoint (left). Tumors were harvested from mice between 3 groups. Tumor volumes (middle) and tumor weights (right) at the endpoint show no significant differences across the three groups. Data are presented as the mean ± SEM, one-way ANOVA followed by Tukey multiple comparison test, n = 4 per group. ns, not significant.
